# EGCG drives gut microbial remodeling-induced epithelial GPR43 activation to lessen Th1 polarization in colitis

**DOI:** 10.1016/j.redox.2024.103291

**Published:** 2024-07-30

**Authors:** Siyan Che, Beibei Qin, Kunfu Wu, Mingzhi Zhu, Han Hu, Can Peng, Zi Wang, Yulong Yin, Yaoyao Xia, Miaomiao Wu

**Affiliations:** a Hunan Provincial Key Laboratory for the Products Quality Regulation of Livestock and Poultry, College of Animal Science and Technology, Hunan Agricultural University, Changsha, Hunan, 410128, China; b Department of Hematology, The Second Xiangya Hospital of Central South University; Molecular Biology Research Center, Center for Medical Genetics, School of Life Sciences; Hunan Province Key Laboratory of Basic and Applied Hematology, Central South University, Changsha 410011, China; c Key Laboratory of Tea Science of Ministry of Education, National Research Center of Engineering Technology for Utilization of Functional Ingredients from Botanicals, Hunan Agricultural University, Changsha, 410128, China; d Institute of Apicultural Research/State Key Laboratory of Resource Insects, Chinese Academy of Agricultural Sciences, Beijing, 100093, China; e Institute of Subtropical Agriculture, Chinese Academy of Sciences, Changsha, 410125, China; f College of Animal Science and Technology, Southwest University, Chongqing, 400715, China

**Keywords:** Inflammatory bowel disease, Th1 cells, EGCG, GPR43, Short chain fatty acids

## Abstract

Modulation of immune microenvironment is critical for inflammatory bowel disease (IBD) intervention. Epigallocatechin gallate (EGCG), as a natural low toxicity product, has shown promise in treating IBD. However, whether and how EGCG regulates the intestinal microenvironment is not fully understood. Here we report that EGCG lessens colitis by orchestrating Th1 polarization and self-amplification in a novel manner that required multilevel-regulated intestinal microecosystem. Mechanistically, EGCG activates GPR43 on IEC to inhibit Th1 polarization dependently of short chain fatty acid (SCFA)-producing gut microbiota. Inhibition of GPR43 activity weakens the protective effects of EGCG on colitis development. Moreover, we confirm that fecal SCFAs and/or intestinal GPR43 are limited in patients with colitis and are correlated with Th1 cell number. Taken together, our study reveals an intestinal microenvironment-dependent immunoregulatory effects of EGCG in treating IBD and provides insight into mechanisms of EGCG-based novel immunotherapeutic strategies for IBD.

## 1 Introduction

Inflammatory bowel disease (IBD) has been characterized by an imbalance in intestinal flora, mucosal damage, impairment of barrier function, and immune cell infiltration [1]. Notably, it is primarily confirmed some main immune cell types, including CD3^+^ T cells [2], macrophages [3], B cells [4], and a few microbiota (e.g. *Ruminococcus* and *Bacteroides*) [5] are involved in the pathogenesis and therapeutic effects of IBD [6]. However, clinical implication of more responding immune subsets and effective responding microbe populations, and their interplay in the gut microenvironment still remain largely unknown.

To date, a series of clinical targeted agents include corticosteroids, immunomodulators, and monoclonal antibodies are developed to intervene inflammatory/trafficking pathways via targeting immune cells in inflammatory microenvironment. Nevertheless, up to 40 % patients do not respond to initial treatment, and half initial responders show loss of clinical response over time [7]. Meanwhile, induction of microbial factors contributes to biological therapy resistance [8,9]. For example, disturbances in *Lachnospiraceae* and *Ruminococcaceae* families have failed to respond to anti-TNF therapy [10]. Additionally, TNF inhibitors cause induction of IL-23 and paradoxical psoriasis-like lesions in IBD patients [11]. These drawbacks highlight the unmet need for uncovering more novel therapeutic approaches (e.g., natural compound) with durable response/low resistance/toxicity that regulate cooperatively inflammatory microenvironment in IBD.

Natural polyphenols have attracted great interest due to their health-promoting properties and are present as main components in various foods and medicinal plants. Epigallocatechin-3-gallate (EGCG) is the only one of commonly consumed natural polyphenols of green tea that have a wide-range of pharmacological and therapeutic properties, including antioxidant, anti-diabetic, anti-inflammatory, and immunomodulatory activities against autoimmune disorders [[12], [13], [14]]. Recently, EGCG has been reported to possess potent anti-cancer effects via modulating immune response in the tumor microenvironment [15,16]. Emerging evidence has also uncovered important links between EGCG and immune cells in regulating autoimmune diseases, such as multiple sclerosis [17]. Although EGCG has been proved to regulate some major populations of immune cells in IBD, the specific responding subtypes that interact with the certain microenvironment and downstream immune molecular effectors of EGCG remains unclear.

In this study, we provide proof-of-concept evidence that EGCG-dependent gut microbiota reprogramming dictates Th1 cell polarization and amplification in the context of DSS-induced colitis. Using fluorescence-activated cell sorting, RNA-sequencing, 16S rDNA gene sequencing, metabolomics, as well as dynamic docking techniques, we uncover that EGCG inhibits the transition of naïve CD4^+^ T to Th1 polarization and Th1 self-amplification by affecting short chain fatty acids (SCFAs)-producing microbes and G-protein-coupled receptor 43 (GPR43) in intestinal epithelial cells (IEC). Importantly, we verify this connection in the cohort of patients with IBD. Collectively, these aforementioned findings unveil an intestinal microenvironment-mediated protective efficacy of EGCG on the pathogenesis of IBD.

## 2 Materials and methods

### 2.1 Animals

C57BL/6J mice (8-week-old males) were purchased from SJA Laboratory Animal Center (Changsha, China). The animals were acclimatized for 7 days and housed in individually ventilated cages in dedicated vivarium with free access to food and water. A maximum of 5 mice were housed per cage, at a regular ambient room temperature (65–75 °F, or 18–23 °C), 40–60 % humidity, and a 12/12 h light cycle. All experiments used mice with the same age and gender for individual experiments. All experimental protocols were approved by the Institutional Animal Care and Use of Committee of Hunan Agricultural University.

### 2.2 Cell lines

Human colonic epithelial cells (HCoEpiCs) and immortalized mouse colonic epithelial cells (MCoEpiCs) were purchased from ScienCell Research Laboratories, Inc. (Carlsbad, CA) and Cellverse Bioscience Technology Co., (Shanghai, China), respectively, and were cultured in Human Epithelial Cell Basal Medium (ScienCell Research Laboratories, Inc., USA) and iCell Mouse Colonic Epithelial Cell Complete Medium (Cellverse Bioscience Technology Co., China), respectively, according to the manufacturers' instructions.

### 2.3 *In vitro* culture models

Epithelial cells (HCoEpiCs or MCoEpiCs) were plated in a 6-well plate at a density of 1 × 10^6^ cells/well, and cultured in Colonic Epithelial Cell Medium. The medium was changed every two days until the cells are fully attached to the culture plates. When cells reached 80 % of the culture dish, they were then subjected to fecal fermentation with EGCG. The *in vitro* fecal fermentation of catechins was performed according to the methodology of Liu et al. [18]. Briefly, freshly passed feces obtained from mice fed EGCG (80 mg/kg/day for 21 days) were immediately transferred to an anaerobic chamber (4 % H_2_, 15 % CO_2_, and 81 % N_2_; Bactron, Cornelius, OR) and mixed with a standard ileal efflux medium at a ratio of 1:40 (w/v). A homogeneous mice fecal suspension obtained by filtration, then 0.9 mL of fecal suspension was separated and spiked with solutions of EGCG in water at a final catechin concentration of 1 mmol/L. The mixtures were then incubated at 37 °C in the anaerobic chamber for 48 h. The 0.1 mL fermentation supernatant was used to incubate colonic epithelial cell with or without GLPG for 24 h, then treated with 2.5 % DSS for 24 h. New cell medium was added after incubation medium removal, and the cells were cultured for another 24 h. These cell culture media were collected for T cell culture. Meanwhile, mouse naïve T cells were activated with anti-CD3 and anti-CD28, followed by addition of anti-IL4, recombinant IL-2, and IL-12 proteins for Th1 polarization or proper treatments shown in Fig. 2, Fig. 5. Phytohemagglutinin-L (Roche, 11249738001) with a working concentration of 1 μg/mL was added to the isolated human T cell culture medium to maintain cell differentiation and metabolism shown in Fig. 6 and S7. T cells were treated with 0.5 % (w/v) DSS for 2 h. Protein transport inhibitor (BD Pharmingen, 555029) was added for 4 h before collecting T cells to detect intracellular factors.

### 2.4 DSS-induced colitis model

Mice were gavaged with EGCG (80 mg/kg body weight, with purity ≥95 %, obtained from Sigma-Aldrich) for 21 or 24 days, and the remaining mice received an equal volume of normal saline (200 μL). To induce colitis, mice were given 2.5 % (w/v) DSS (MP Biomedicals) in drinking water for 5 days, and allowed for 2 days recovery with normal drinking water.

During the DSS administration period, animals were monitored daily for weight loss, and observed for stool consistency and rectal bleeding to evaluate disease activity index (DAI). At the end of the experiment, mice were fully anesthetized and sacrificed by dislocating the cervical vertebrae. The abdominal cavity was opened longitudinally, mouse livers were washed with cold phosphate buffered saline (PBS), and weighed. Colons were carefully dissected and their lengths were measured, and colonic stools were collected by sterile cryovial. Immediately after removal, 0.5 cm colon specimens were fixed for morphological and immunofluorescence analysis, 1 cm colon were fresh-frozen in liquid nitrogen and stored at −80 °C until use, and the rest colon specimens were stored in cold Hanks’ balanced salt solution (HBSS) containing 5 % fetal bovine serum (FBS) for subsequent flow cytometric analysis.

### 2.5 Antibiotic treatment of mice

To abolish the role of the intestinal microbiota in EGCG alleviating colitis, mice received a volume of 10 μL/g body weight of drinking water, supplemented with an antibiotic cocktail (ABX) of 0.1 mg/mL Amphotericin-B, 10 mg/mL Ampicillin, 10 mg/mL Neomycin trisulfate salt hydrate, 10 mg/mL Metronidazole, and 5 mg/mL Vancomycin hydrochloride delivered with a stainless-steel tube for 2 weeks. Animals displayed successful depletion of their cultivable aerobic and anaerobic fecal microbiota [19]. Samples were collected at day 14.

### 2.6 GLPG0974 treatment of mice

To study the role of SCFA receptors, potent FFAR2 (GPR43) antagonist, GLPG0974 (HY-12940, MCE, Monmouth) was orally dosed as a single esophageal gavage at 10 mg/kg to mice before the DSS intervention for 24 days (once every three days) [20]. Colitis parameters were recorded and samples were collected at the end of the experiment.

### 2.7 Histological score analysis

Formalin-fixed colon tissues were embedded in paraffin and cut into 4 μm sections. Then sections were stained with Hematoxylin & Eosin (H&E). Pathology scores were determined according to a previous study [21]. Briefly, scoring was conducted in a blinded manner by a pathologist based on villi blunting, crypt loss, and regeneration, epithelial cell apoptosis, immune cell infiltrates, and mucosal ulceration of lamina propria (0.5, focal and rare; 1, focal and mild; 2, diffuse and mild; 3, diffuse and moderate; 4, diffuse and severe).

### 2.8 Microbiota analysis based on 16S rRNA sequencing

Fecal samples were collected from individual mice and total microbial genomic DNA was extracted using QIAamp PowerFecal Pro DNA Kit (Qiagen, Germany) according to the manufacturer's instructions. The microbiome samples were analyzed using barcoded high-throughput amplicon sequencing of the bacterial 16S rRNA gene. Briefly, the concentration and integrity of DNA were assessed using Nanodrop (Thermo Fisher Scientific). Diluted DNA (1 ng/mL) was then used to amplify the V4 hypervariable region of the 16S rRNA gene with barcoded primers (515 F, 5′-GTGCCAGCMGCCGCGGTAA-3′, 806 R, 5′-GGACTACHVGGGTWTCTAAT-3′) and Phusion® High-Fidelity PCR Master Mix with GC Buffer (New England Biolabs, USA). PCR amplification products were purified, and DNA was used to prepare sequencing libraries using TruSeq® DNA PCR-Free Sample Preparation Kit (Illumina). Library quality was evaluated on Qubit 2.0 Fluorometer (Thermo Fisher Scientific, USA). Purified PCR products were then sequenced using the Illumina NovaSeq platform (Thermo Fisher Scientific, USA) with 250 bp paired-end reads.

Raw sequencing data was analyzed using the QIIME2 platform (version 2020.2). Raw reads were quality filtered, assembled, and chimeric sequences were removed based on Silva database. Sequences were assigned to operational taxonomic units (OTUs) using the UCLUST algorithm68 with a sequence identity threshold of 96 %. Taxonomic assignments of each operational taxonomic unit were made by similarity searching against the publicly available 16S (RDP version 10.27 and CORE update September 2, 2012) and the NCBI genome database using the GLSEARCH program. The data were rarefied to 10,000 sequences per sample, as determined by the rarefaction curves. Relative abundances of the community members were determined using the rarefied data. UniFrac analysis was performed as described previously [22]. Alpha, beta diversity, and environmental factor correlation (Spearman analysis) were calculated with QIIME (Version 1.7.0) and displayed with R software (Version 2.15.3). Principal coordinates analysis (PCoA) plots were displayed by WGCNA package, stat packages, and ggplot2 package in R software (version 2.15.3). Illumina MiSeq sequencing, processing of sequencing data, and bioinformatics analysis were performed by Beijing Novogene Bioinformatics Technology Co., Ltd. (China).

### 2.9 Metabolite profiling analysis

The fecal samples or supernatant were used to perform metabolomics assay using a gas chromatographic (GC) system equipped with a flame ionization detector (FID) to detect levels of short chain fatty acids (SCFAs), including acetic acid, propionic acid, butyric acid, isobutyric acid, isovaleric acid, and valeric acid). Samples were weighted for 1 g, added with 5 mL ddH_2_O, and mixed thoroughly on a vortex mixer, and shaken on an oscillator for 30 min. Samples were then incubated overnight at 4 °C. After centrifugation (10,000 r/min, 10 min, 4 °C), the supernatant 1 was transferred into a 10 mL cuvette. Subsequently, supernatant 2 was harvested into the same cuvette to get total supernatant, followed by the pellet was collected, dissolved with 4 mL ddH_2_O, and put in an oscillator for 30 min. The total supernatant was spun down for 15 min at 12,000 rpm. Then, the supernatant was mixed with 25 % (v/v) metaphosphoric acid at a volume ratio of 9:1 (v/v), allowed to stand for 3–4 h at RT (centrifuge again at 12,000 rpm for 15 min if pellet appeared). The resulting supernatant was filtered through a 0.22 μm disposable membrane and transferred into an N10149 automatic sampler (Agilent Technologies, USA). Subsequently, the standard curve was created for quantification of SCFAs by GC system. Stock solutions of different SCFAs (Sigma, USA) were prepared, and gradient of 20 μL, 50 μL, 100 μL, 200 μL, 300 μL, and 500 μL were mixed with 100 μL 25 % (v/v) metaphosphoric acid, then ddH_2_O was added to reach a final 1000 μL volume.

Chromatographic analysis was performed on a DB-FFAP column (30 m × 250 μm × 0.25 μm; Agilent Technologies, USA) with an FID. High purity nitrogen (99.999 %) was applied as carrier gas at a constant flow rate of 0.8 mL/min, and high purity hydrogen (99.999 %) was used as the auxiliary gas. The determination method was set as follows. The FID detector temperature was maintained at 280 °C, the injection temperature was 250 °C, and split liner was applied as intermediary mixing chamber for the sample to vaporize before encountering the stationary phase of the column. The split injection ratio was set 50:1 and the injection volume were 1 μL. Initial column temperature was 60 °C, increased to 220 °C at a rate of 20 °C/min and maintain for 1 min.

SCFAs were identified by the times of retention in comparison with analytical standard solutions. Agilent OpenLab CDS 2.6 software was used to integrate the areas of each peak to get the C value, corresponding to respective SCFA. Concentrations of SCFAs were calculated based on the standard curve using the formula as follows: X = C*V*10/9M, where X represents SCFA values (μg/g), C represents concentrations in the sampler (μg/mL), V represents volume (mL), M represents sample weight (g), 10/9 represents metaphosphoric acid volume conversion ratio.

### 2.10 RNA sequencing

Colon samples were collected and snap-frozen. RNA extraction, quality verification, library preparation, and sequencing were performed by Beijing Novogene Bioinformatics Technology Co., Ltd. (China). Briefly, tissue total RNA was extracted with Trizol (Thermo Scientific, USA). RNA quantity and purity were analyzed with Bioanalyzer 2100 and RNA 6000 Nano LabChip Kit (Agilent, USA) with RIN number >8.0. The template fragmented mRNA, obtained by enriching total RNA with Oligo (dT) magnetic beads, was randomly interrupted and reverse transcribed into cDNA. For cDNA library constructions, approximately 5 μg of total RNA per sample was used for the RNA sample preparations. The library for sequencing was generated using an Illumina TruSeq RNA Sample Preparation Kit (Illumina, San Diego, USA). Transcriptome sequencing was carried out on an Illumina novaseq6000 platform. Raw data of sequencing were uploaded to https://magic.novogene.com/customer/main#/login for subsequent analysis. Differentially expressed genes (DEGs) by statistics with an adjusted *p*-value <0.05 and fold change >1.5 were set as the threshold values for KEGG over-representation analysis and gene ontology (GO) over-representation analysis.

### 2.11 ELISA

Colon tissues were homogenized and centrifuged, and supernatant was collected. Culture supernatants from cell culture were also collected. Levels of cytokines (Mouse IFN-γ, KE10094; mouse IL-12, KE10014; human IFN-γ, KE00063; human IL-12, KE00019) were measured by ELISA commercial kits following the protocols (Proteintech Group Inc., USA).

### 2.12 Molecular docking

The protein structure of mouse GPR43 used for docking was obtained from the Alphafold database (https://alphafold.ebi.ac.uk/). Structures of acetate, propionate, and EGCG molecules were downloaded from the PubChem database. Molecular docking was conducted using AutoDock Vina 1.1.2 software, and solvent molecules and ligands were removed by PyMol 2.5 software. Based on previous research [23], arginine 180 (Arg 180) was set as the docking center and limited the molecular search range using a 40 ✕ 40 docking box. Following the docking process, Protein-Ligand Interaction Profiler (PLIP) was employed to analyze the non-covalent interactions at the atomic level within the protein-ligand complex. The global search exhaustiveness was set to 32, and all other parameters were maintained at their default settings. The highest-scoring docked conformation output was considered the active pose, and the docking conformation was visually analyzed using PyMol 2.5.

### 2.13 RNA extraction and qRT-PCR

Total RNA was extracted using TRIzol reagent Trizol™Up Kit (Invitrogen, USA), and conducted according to the manufacturer's protocol. Then cDNA was synthesized using a RevertAid First Strand cDNA Synthesis Kit from Thermo Fisher Scientific. Real-time PCR analysis was performed according to TB Green™ Premix Ex Taq™ (Tli RNaseH Plus, RR420A; Takara Bio, Japan). Primers were designed in accordance with the principle of PCR primer design and β-actin was used as the internal control gene. All primers were purchased from Sangon Biotech. Details were listed in Table S1.

### 2.14 Protein extraction and western blotting

Western blotting assay was conducted according to the well-established standard method [24]. Briefly, frozen tissues were cut into pieces, and nuclear and cytoplasmic proteins were extracted using the NE-PER™ Nuclear and Cytoplasmic Extraction Reagents (Thermo Fisher Scientific, USA). Protein concentration was detected by bicinchoninic acid (BCA) protein assay kit (Beyotime, China) according to the manufacturer's manual. About 15–20 μg of total protein were loaded into each well. After separating by 10 % separating gels, proteins were transferred onto a PVDF membrane (BioRad, USA), and blocked with 5 % BSA Tris-Tween-buffered saline buffer (TBST) for 1 h at RT. Then transferred membranes were incubated with the primary antibodies overnight at 4 °C. The primary antibodies were listed in Table S1. Subsequently, the HRP-conjugated secondary antibodies (anti-mouse 1:5000, Proteintech; anti-rabbit 1:6000, Abcam; anti-goat 1:10000, Santa Cruz) were incubated for 2 h at RT. Finally, the membrane was developed with ECL chemiluminescent reagent (Thermo Fisher Scientific, USA), and the target protein bands were detected using chemiluminescence imaging analyzer ChemiDoc (BioRad, USA). The grayscale values were calculated using Image J software.

### 2.15 Immunofluorescence assay

After washing twice with PBS, cells were fixed, permeabilized, and blocked using quickblockTM blocking buffer for immunostaining (Beyotime, China). Then samples were incubated for 1 h at 37 °C with primary antibodies. Rabbit anti-human/mouse GPR43 (Santa Cruz, sc-32906) were used at 1:300 and 1:500. After incubating primary antibodies and washing, the slides were then incubated with secondary antibody (anti-rabbit Alexa Fluor 488 Conjugate, Absin, abs20025, 1:500 diluted in 5 % goat serum) for 1 h. Images were acquired by a confocal fluorescence microscope (Leica, Germany).

### 2.16 Cell isolation

The colon was washed with pre-chilled HBSS/Hepes buffer [1 × HBSS supplemented with Hepes (1.5 mM)]. Tissues were cut longitudinally and into 1- to 2-cm pieces, which were incubated in HBSS/EDTA buffer [1 × HBSS supplemented with EDTA (5 μM)] for 2 × 20 min at 37 °C on an oscillator at 500 rpm. After each incubation, intestine pieces were filtered via a 150-μm nylon strainer and the flow-throughs were collected as fraction I. Subsequently, tissue pieces were rinsed with HBSS/Hepes and digested in HBSS supplemented CaCl_2_ (1 mM), collagenase VIII (0.3 mg/mL), and DNase I (25 μg/mL) for around 45 min at 37 °C on a shaker at 300 rpm. The resulting suspension was filtered via a 100-μm nylon strainer and the flow-through was collected as fraction II. Fraction I and II were combined and further purified by density gradient spinning using (40 %/80 %) Percoll solutions. The purified cells were used as intestinal immune cells. Single-cell suspensions of spleens were generated by mashing through 100-μm cell strainers and sorted as mouse CD4^+^ T cells with proper markers. To sort CD4^+^ T cells in human peripheral blood mononuclear cells (PBMC), blood from healthy donor was stained after directly separation with Ficoll (Absin, abs930). Red blood cell lysis on splenocytes and blood cells was performed with BD Pharm Lyse™ Lysing Buffer following the manufacturer's instructions. Isolated cells were resuspended in 1 mL cold FACS buffer and viable cells were counted using trypan blue exclusion for further staining.

### 2.17 Th1 cell polarization

CD44^-/low^CD62L^high^CD4^+^ T cells were purified from mouse spleen cells by sorting with FACS Melody™ (BD Bioscience) after staining with anti-CD44 (IM7, BD Pharmingen), anti-CD62L (MEL-14, BD Pharmingen), and anti-CD4 (RM4-5, BD Pharmingen) antibodies, and were used as mouse naïve CD4^+^ T cells. CD45RA^+^CCR7^+^CD4^+^ T cells were sorted similarly from PBMC of healthy volunteers after staining with anti-CD45RA (2D1, BD Pharmingen), anti-CCR7 (3D12, BD Pharmingen), and anti-CD4 (RPA-T4, BD Pharmingen) antibodies, and were applied as human naïve CD4^+^ T cells. To polarize T cells into Th1, isolated naïve CD4^+^ T cells were plated in a 12-well plate pre-coated with anti-CD3 (145-2C11, BD Pharmingen) and anti-CD28 (37.51, BD Pharmingen) or pre-added with phytohemagglutinin-L (PHA-L, Roche, Switzerland) for activation, then cells were cultured with media containing 10 ng/mL IL-12 (Biolegend), 10 ng/mL IL-2 (BioLegend), and 10 μg/mL anti-IL-4 (11B11, BioLegend) for three days. Subsequently, EGCG (0.5, 1, and 2 μM) or the fermentation supernatant was added to cells for co-incubation, and the functional factors of Th1 cells were detected. Key resources are listed in Table S1.

### 2.18 Flow cytometry

Flow cytometry analysis and cell sorting were performed in line with standard procedures using the antibodies described in Table S1. Fixable Viability Stain were used to identify dead cells, and FSC-A versus FSC-H/W scatterplots were used to identify cell aggregates that were excluded from analysis. Intercellular staining was conducted using Cytofix/Cytoperm™ Fixation/Permeabilization Kit or Transcription Factor Buffer Set (BD Pharmingen, USA) according to the manufacturer's instructions. Before flow cytometry cell analyzing/sorting, cells were stained with fluorophore-coupled antibodies. To detect intestinal cell subpopulations, isolated cell suspensions were prepared in FACS buffer and stained with markers to identify the specific populations. Flow cytometry analysis was performed using an FACSCelesta™ Cell Analyzer (BD Biosciences, USA), and cell sorting was performed on FACSMelody™ Cell Sorter. Results were analyzed using FlowJo™ software v10 (BD Biosciences, USA). Single stains were used to set appropriate gates for each fluorochrome and respective sample type. Bad events were excluded by FlowAI. Data were quantified as frequencies of specified parent populations or total cells.

### 2.19 Human SCFAs and bulk RNA-seq data

Untargeted metabolic data for SCFA concentrations in human stools (including non-IBD, Crohn's disease (CD), and Ulcerative colitis (UC) patients) were obtained from Project PR000639 in the Metabolomics Workbench (http://www.metabolomicsworkbench.org) [25]. Bulk RNA-sequencing data for biopsies collected 10 cm from rectum are is accessed through GEO Series accession number GSE111889. Fecal SCFA and biopsy RNA-seq data were originated from same patient in week 0, as they were diagnosed as non-IBD, CD, or UC, and had not been medically treated. Th1-related genes including IL12A, IL12B, IL12RB1, IL12RB2, LTA, IFNG, CXCR3, TBET, STAT1, and STAT4 were scored based on gene set variation analysis (GSVA) enrichment score.

### 2.20 Quantification and statistical analysis

The significance of the differences between two different groups was evaluated by unpaired two-tailed Student's t-test; one-way ANOVA (with Tukey's multiple-comparison tests) was used for comparisons between more than two groups, if the data were in Gaussian distribution and had equal variance. The correlation analysis was performed using the Spearman's correlation test. *P* values less than 0.05 were considered significant. Statistical analysis was done by GraphPad Prism (8.0, GraphPad, USA). Data were represented as means ± SEM, *P < 0.05; **P < 0.01. *In vitro* data were from at least three samples and were representative from one of three experiments performed.

## 3 Results

### 3.1 EGCG decreases intestinal CD4^+^ Th1 cell numbers in mice with ulcerative colitis

**Fig. 1:**
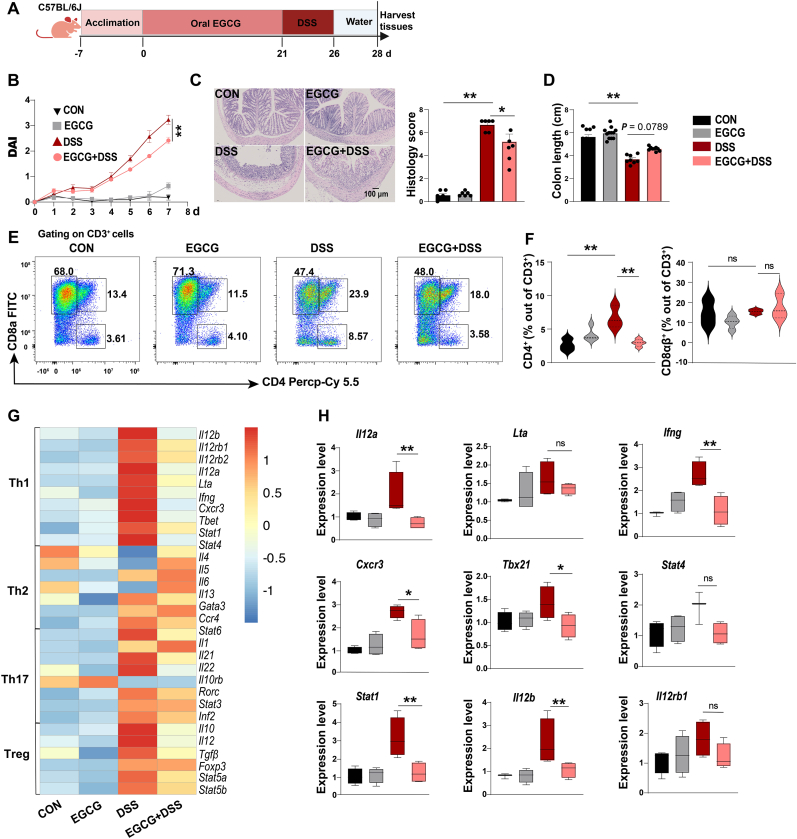
**EGCG reduces gut Th1 cell numbers in DSS-induced colitis.** (A) Experimental scheme for (B–H). C57BL/6 mice were administrated 2.5 % (w/v) DSS in drinking water for 5 days after they were treated with 80 mg/kg/day EGCG via oral gavage for 21 days, then allowed to recover for 2 days using normal drinking water. (B) Body weight, gross bleeding, and stool consistency were recorded daily to calculate disease activity index (DAI). n = 8–12. (C) Representative images of colonic histopathology by hematoxylin and eosin staining and pathological scoring. Scale bar, 100 μm, n = 6 (D) Colon length was measured. n = 8–12. (E and F) Colonic T cell subtypes were detected by flow cytometry after colon immune cells were isolated, and CD4^+^ and CD8^+^ T cells were quantified using FlowJo, gating strategy (E) and cell subpopulation ratios were obtained (F). Two to three mice were pooled for one sample, data are one representative of two independent experiments. n = 3. (G) RNA-seq analysis for colon tissue included Th cell differentiation-related transcriptome heatmap. n = 6–10. (H) mRNA expression of factors that promote Th1 differentiation in colon tissue were detected by qRT-PCR. n = 6–8. Data are presented as mean ± SEM. Statistical significance was determined using one-way ANOVA, followed by Tukey's multiple-comparison tests or Student's t-test. *P ≤ 0.05, **P ≤ 0.01. See also Figure S1.

EGCG has shown therapeutic effects against various metabolic and autoimmune disorders by modulating the T cell compartment. However, its impact on the polarization of CD4^+^ and/or CD8^+^ T cell subsets in the context of colitis remains unclear. It should be mentioned that the EGCG dose was selected based on the level of exposure falling within the upper range (300–866 mg/day) of high-level consumers of green tea infusions in the EU adult population [26]. The animal equivalent dose was calculated based on body surface area by multiplying the human dose (mg/kg) by the Km ratio (Km = 12.3) [27]. We evaluated dose-response relationship for EGCG (10, 20, 40, 80, 100 mg/kg/day) in preliminary experiments (data not shown) and found that mice pre-treated with oral gavage of 80 mg/kg EGCG for 21 days might show the optimal therapeutic potential for lessening colitis (Fig. 1A). Our results showed that although EGCG pre-treatment initiated from colitis onset on day 5 had little effect on body weight changes (Fig. S1A), it significantly reduced the severity of DSS-induced colitis as measured by decreased DAI scores (Fig. 1B) and improved histological parameters, such as reduced cell infiltration and damage to colonic mucosa (Fig. 1C). Additionally, EGCG treatment also tended to prevent colon length reduction in DSS-induced mice (Fig. 1D).

**Fig. 2:**
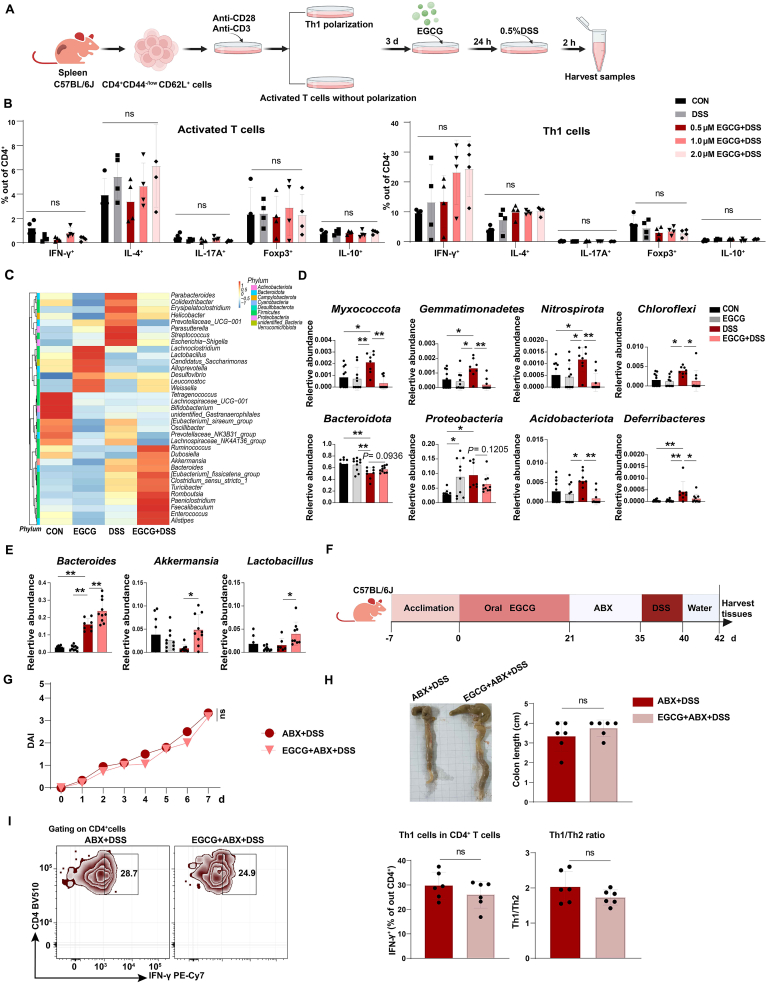
**EGCG indirectly regulates the Th1 subtype via microbes.** (A) Experimental scheme of EGCG directly treatment for activated T and Th1 cells in (B). Naïve CD4^+^ T cells from mouse spleens were isolated, activated *in vitro* using anti-CD28 and anti-CD3, and induced Th1 polarization. Cells were then stimulated with 0.5 % (w/v) DSS after incubation of EGCG with different concentrations (0.5 μM, 1.0 μM, and 2.0 μM) for 24 h. (B) Effects of direct EGCG treatment on cell function-related factors of activated T and polarized Th1 cells were determined by flow cytometry. Data are one representative of two independent experiments. n = 4. (C–E) Mice were orally gavaged with 80 mg/kg/day EGCG for 21 days, then given drinking water containing 2.5 % (w/v) DSS for 5 days, followed by 2 days of recovery. 16S rDNA sequencing for colon feces was performed via the 454 FLXtitanium system and sequences were analyzed using QIIME software. Heatmap showed differences of top 35 microbial gene enrichment for different treatments (C). Bacterial difference at the phylum (D) and genus (E) levels among groups were analyzed, bacteria with significant differences were listed. n = 8–10. (F) Experimental scheme for antibiotic treatment in (F–I). Mice were administered EGCG at 80 mg/kg/day orally for 21 days, then treated with an antibiotic cocktail (ABX, a volume of 10 μL/g body weight of drinking water supplemented with 0.1 mg/mL Amphotericin-B, 10 mg/mL Ampicillin, 10 mg/mL Neomycin Trisulfate salt hydrate, 10 mg/mL Metronidazole, and 5 mg/mL Vancomycin hydrochloride) for 14 days. The mice were then given water containing 2.5 % (w/v) DSS for 5 days, allowed for 2 days recovery with normal water (F). (G and H) Daily DAI (G) during the experiment were recorded. n = 8–10. At the end of the experiment, the mice were sacrificed, and colon length (H) were measured. n = 6. (I) Th1 cell subsets were detected and quantified by flow cytometry after colonic immune cells were isolated. Data are one representative of two independent experiments. n = 6. Data are shown as mean ± SEM. Statistical significance was determined using one-way ANOVA, followed by Tukey's multiple-comparison tests or Student's t-test. *P ≤ 0.05, **P ≤ 0.01. See also Figure S2–S4.

To explore the impact of EGCG treatment on the intestinal microenvironment in colitis, we performed a comprehensive analysis of various immune cells, including CD4^+^ T, CD8αβ^+^ T, CD79a^+^ B cells, and F4/80^+^ macrophages, using flow cytometry (Fig. S1B). Similar to a previous study [28], EGCG pre-treatment led to slightly increased in macrophages compared to the untreated DSS group (Fig. S1C). Interestingly, EGCG pre-treatment resulted in a remarkable reduction in the number of CD4^+^ T cells, while no significant differences were observed in the other cell populations (Fig. 1E and F, and S1C). Further analysis using RNA-seq revealed that EGCG had the most significant impact on the expression of marker genes of Th1 cells, including *Il12a*, *Ifng*, *Cxcr3*, *Tbx21*, *Stat1*, and *Il12b* (Fig. 1G and H). Collectively, our results demonstrates that the Th1 cell subset might be one of the key responding immune cell subpopulations to EGCG treatment in colitis mice, although the additional experiments (e.g. Th1 cell depletion) are still needed in the future.

### 3.2 EGCG regulates Th1 population that relies on reshaping microbiota

To elucidate the mechanisms by which EGCG regulates the Th1 subset, we isolated primary CD4^+^ effector cells from mice and activated them under Th1-polarization conditions. Th1 cells were then treated with EGCG followed by 0.5 % DSS (Fig. 2A). Our flow cytometry results showed that EGCG treatment did not affect the expression of representative mouse T cell markers IFN-γ, IL-4, IL-17A, Foxp3, and IL-10 in either activated T or polarized Th1 cells compared to the corresponding DSS groups (Fig. 2B–S2A, and S2B). This indicates that direct treatment with EGCG *in vitro* did not affect effector T cell functions, the polarization of Th1 and other T cell subsets or the amplification of effector T/Th1 cells under the inflammatory condition. Given the critical role of microbiota-immune interactions in the pathogenesis of colitis, we next investigated the impact of EGCG on the gut microbiota composition. PCoA analysis revealed a dramatic separation in the clustering of microbial community structures between the EGCG-treated and control groups (Fig. S3A). Additionally, Shannon diversity analysis showed that EGCG treatment reduced the microbial diversity under normal or colitis conditions (Fig. S3B). Further analysis of the microbial ecology and composition revealed that DSS treatment alone led to an enrichment of pathogenic bacteria (Figs. S3C–S3E), such as *Escherichia−Shigella, Streptococcus, Helicobacter, Erysipelatoclostridium*, and *Colidextribacter* (Fig. 2C). In contrast, EGCG treatment significantly inhibited the abundance of these pathogenic taxa. Further, at the phylum level, EGCG markedly enhanced *Bacteroidota* enrichment, a large group of protective gut commensal strains, and decreased the enrichment of *Myxococcota, Gemmatimonadetes, Nitrospirota, Proteobacteria,* and *Acidobacteriota* (Fig. 2D). At the genus level, EGCG intervention increased the abundance of three probiotics: *Lactobacillus*, *Akkermansia,* and *Bacteroides* (Fig. 2E). Interestingly, *Lactobacillus* correlates with energy intake and promotes mucosal formation. Bacteroides is a major group of bacteria that produce gut propionate, and *Akkermansia* produces acetate and propionate [25]. Therefore, our results suggest that EGCG reshapes gut microbiota composition, especially for an enrichment of SCFAs-producing probiotic strains in mice with DSS-induced colitis.

To further investigate the role of microbiota in EGCG's regulation of Th1 cells, we treated mice with an ABX of amphotericin-B, ampicillin, neomycin, metronidazole, and vancomycin in the drinking water after oral EGCG treatment, followed by the induction of DSS colitis and a 2-day recovery period (Fig. 2F). Interestingly, the protective effects of EGCG were abolished in the ABX-treated colitis mice supplemented with EGCG (the EGCG + ABX + DSS group), as indicated by similar DAI scores and colon lengths (Fig. 2G and H). Further analysis of the Th1 cell population revealed that the ABX treatment dramatically abolished the EGCG-mediated reduction in Th1 cells in DSS-induced colitis mice (Fig. 2I–S4A, and S4B). Together, these findings suggest that EGCG's effects on the Th1 cells might be indirectly mediated through the remodeling of the gut microbiota.

### 3.3 EGCG activates colonic SCFA receptor GPR43 to inhibit Th1 polarization

**Fig. 3:**
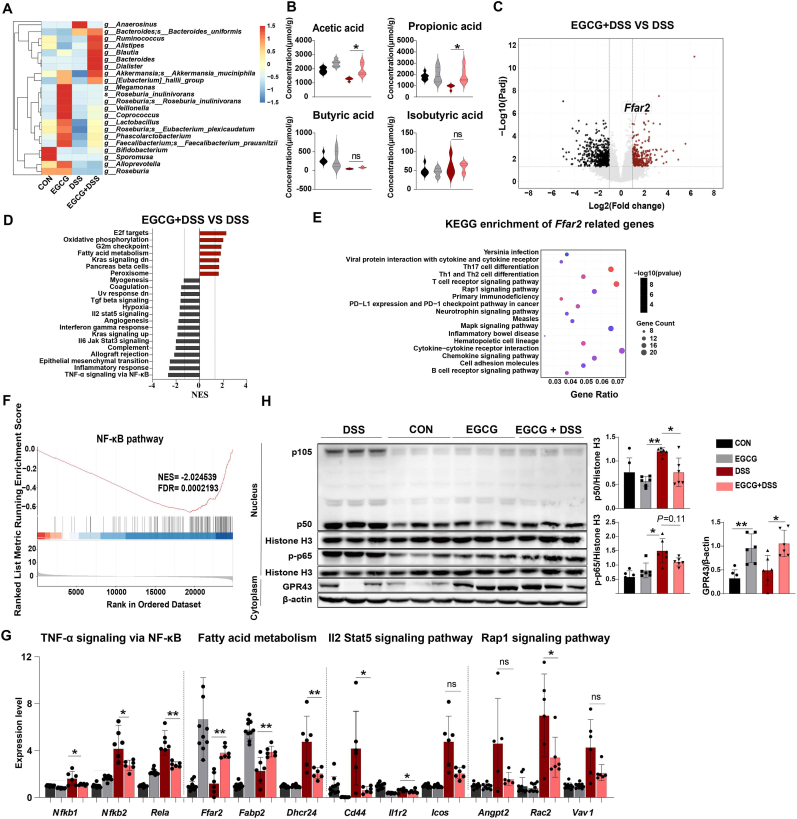
**EGCG activates colonic SCFA receptor GPR43 in mice.** Mice were administrated 2.5 % (w/v) DSS in drinking water for 5 days after they were treated with 80 mg/kg/day EGCG via oral gavage for 21 days, then allowed to recover for 2 days using normal drinking water. (A) The abundance of SCFA-producing bacteria in colon feces. n = 8–10. (B) Fecal concentrations of SCFA. n = 6–8. (C–E) RNA-Seq analysis for colon tissues revealed the DEGs (C) and signaling pathways (D) in the EGCG treated colitis mice compared with the non-treated, as well as KEGG of *Ffar2* related genes (E). n = 8–10. (F) GSEA from RNA-seq analysis for colon tissues showed the significant downregulation of NF-κB in EGCG treated colitis mice. n = 8–10. (G) qRT-PCR results for colon tissues showed gene levels of GPR43 downstream signaling pathways. n = 4–6. (H) Western blotting analysis of NF-κB signaling and GPR43 were determined and quantified. Data are one representative of two independent experiments. n = 3. Data are presented as mean ± SEM. Statistical significance was determined using one-way ANOVA, followed by Tukey's multiple-comparison tests or Student's t-test. *P ≤ 0.05, **P ≤ 0.01. See also Fig. S5.

It has been found that acetate is produced by most enteric bacteria such as *Bacteroide, Ruminococcus, Alistipes, Akkermansia muciniphila, Blautia, Lactobacillu,* and *Bifidobacterium* [29,30]. Propionate-producing bacteria include *Akkermansia muciniphila, Bacteroidetes, Eubacterium hallii* and *Faecalibacterium prausnitzii.* Moreover, *Megamonas and Roseburia inulinivorans* are common butyrate-producing bacteria [31,32]. Consistent with these observations, our analysis revealed that EGCG selectively affected the representative acetate- and propionate-producing bacteria, with a relatively weak impact on the butyrate-producing bacteria under inflammatory conditions (Fig. 3A). Consistent with changes in SCFAs-producing bacteria, high throughput metabolomics further confirmed elevated concentrations of acetate and propionate in colonic contents of EGCG-treated colitis mice (Fig. 3B).

**Fig. 4:**
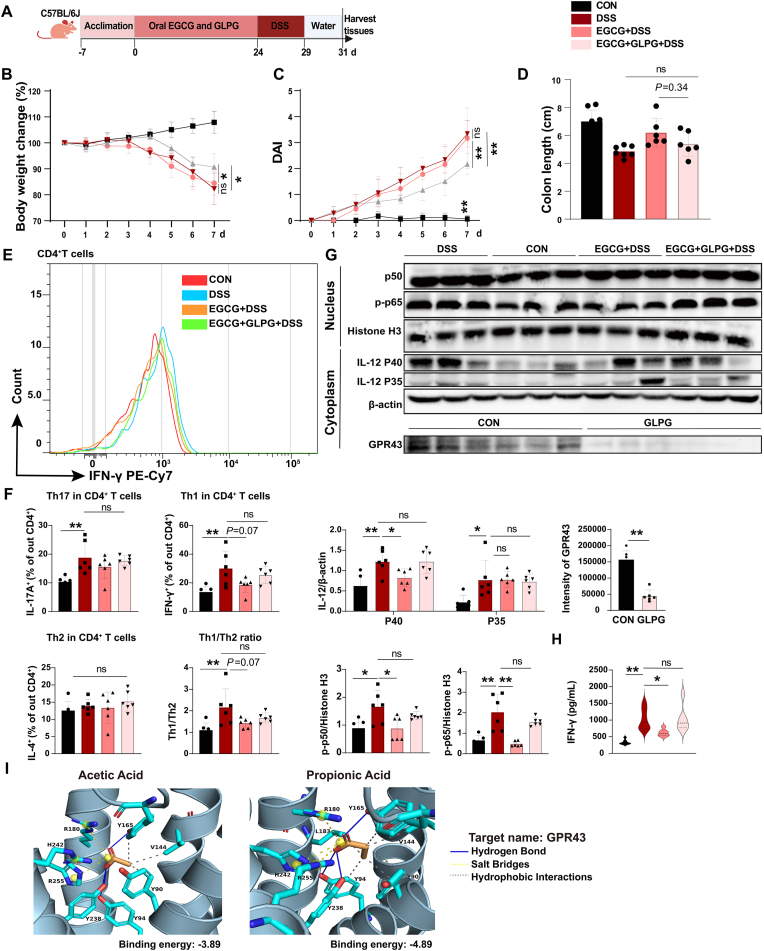
**GPR43 mediates inhibition of Th1 polarization by EGCG.** (A) Experimental scheme for inhibitor treatment in (B–G). Mice were treated with 80 mg/kg/day EGCG for 24 days, and orally gavaged GLPG0974 (GPR43 inhibitor) at 10 mg/kg for every 3 days, then mice were administrated 2.5 % (w/v) DSS in drinking water for 5 days, and allowed to recover for 2 days using normal drinking water. (B–D) Body weight changes (B), disease activity index (DAI) (C), and colon length (D). n = 6. (E and F) T cell subtypes were detected by flow cytometry after colonic immune cells were isolated and stained. CD4^+^IFN- γ^+^ T cells (Th1) were showed (E), Major effector T cells including CD4^+^IL-17A^+^ T cells (Th17), CD4^+^IL-4^+^ T cells (Th1) were quantified after flow cytometry, and Th1/Th2 ratio was calculated (F). n = 6. (G) Expressions of NF-κB signals, IL12, and GPR43 in colon were determined by Western blot. Data are one representative of two independent experiments. n = 3. (H) IFN-γ protein concentration in colon was detected by ELISA. n = 6. (I) Dynamic molecular docking was employed to study interactions and binding affinity between acetate/propionate and GPR43, amino acid residues were predicted. Data are presented as mean ± SEM. Statistical significance was determined using one-way ANOVA, followed by Tukey's multiple-comparison tests or Student's t-test. *P ≤ 0.05, **P ≤ 0.01.

Given that the metabolite-sensor GPR43 (also known as free fatty acid receptor 2, FFAR2) is the receptor for SCFAs, we next explored the effects of EGCG on the expression and activity of colonic GPR43. Comparative transcriptomic analysis of colon tissues from colitis mice supplemented with EGCG or vehicle control revealed a total of 919 differentially expressed genes (DEGs), including 262 up-regulated and 657 down-regulated genes in the DSS-induced group (Fig. S5A). KEGG enrichment analysis showed that the top 20 upregulated genes were enriched in various inflammation-related signaling pathways, such as TLR, JAK-STAT, NF-κB, TNF, and MAPK, and Th cell (Th1, Th2, Th17) differentiation (Fig. S5B). Interestingly, the volcano plot of RNA-seq data showed that EGCG significantly upregulated the expression of *Ffar2,* which encodes the GPR43 protein (Fig. 3C). Further analysis of the key downstream signaling components of GPR43 revealed that EGCG dramatically activated GPR43-mediated pathways, including NF-κB-TNF-α signaling, fatty acid metabolism, Il2-Stat5 signaling, and Rap1 signaling under inflammatory conditions (Fig. 3D and E). Among these, the NF-κB pathway was the most significantly downregulated (Fig. 3F). The altered expression of GPR43 and its key signaling components were further confirmed by qRT-PCR (Fig. 3G) and western blotting analysis (Fig. 3H). Our results showed that EGCG reduced nuclear expressions of p50 (NFKB1) and p-p65 (RELA), while promoting total protein expression of GPR43 with or without DSS treatment (Fig. 3H). Of notes, Spearmen's analysis showed a strong correlation between NF-κB and SCFA pathway-related gene components in the EGCG treatment groups, suggesting that GPR43 signaling is a key downstream effector for EGCG's actions (Fig. S5C). To verify the role of GPR43 in mediating inhibitory effects on Th1 cells in colitis, we used the GPR43 inhibitor GLPG0974 to block GPR43 activity in EGCG-treated mice (Fig. 4A). Interestingly, the ameliorative effects of EGCG on colitis were markedly weakened by GLPG addition (Fig. 4B–D). Furthermore, the proportion of Th1 cell population did not show obvious changes after oral administration of EGCG in DSS-treated mice with GPR43 blockade (Fig. 4E and F). Correspondingly, the EGCG-mediated alterations in GPR43-related signaling components, including decreased nuclear expressions of p-p50 and p-p65, as well as decreased cytoplasmic expressions of IL-12 and IFN-γ levels in the colon, were restored to levels similar to the DSS group upon GLPG intervention in colitis mice (Fig. 4G and H).

**Fig. 5:**
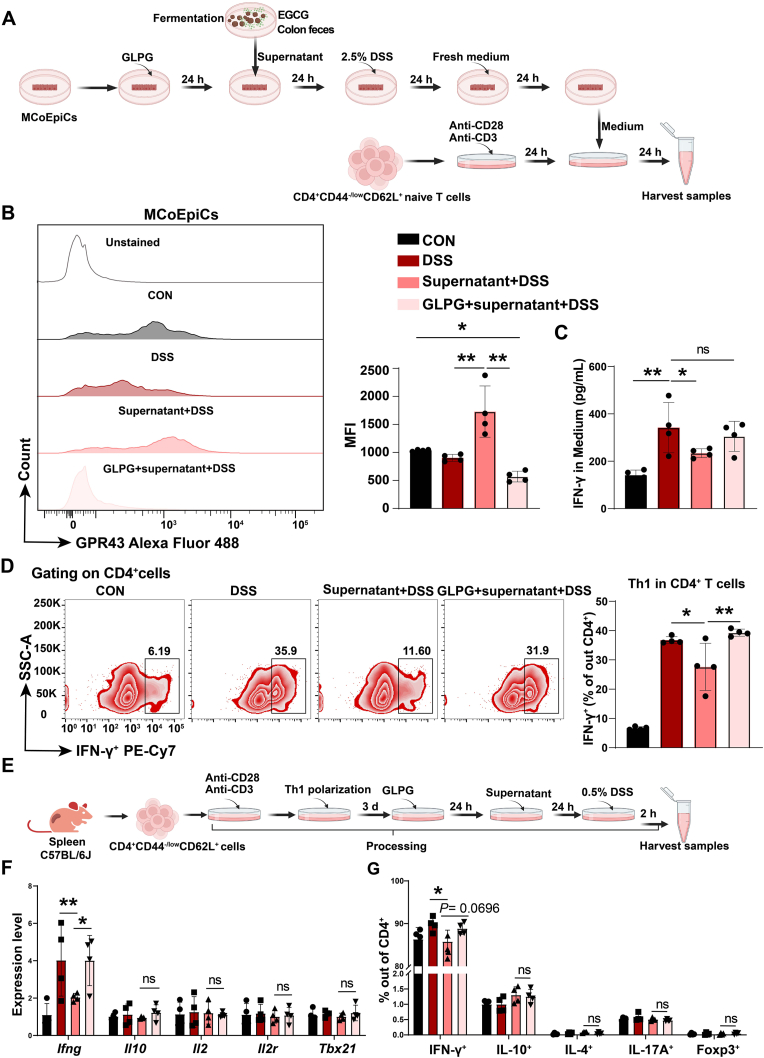
**Epithelial GPR43 is required for the inhibition of EGCG fermentation on Th1 polarization.** (A) Experimental scheme for (B–D). EGCG and mouse colonic contents were fermented, and the fermentations was centrifuged, then concentrated at low temperature and diluted with cell culture medium. The resulting supernatant was used to treat mouse colonic epithelial cells (MCoEpiCs) for 24 h after epithelial cells were treated with GLPG for GPR43 inhibition. Then culture medium was refreshed and followed by 2.5 % (w/v) DSS treatment. The culture medium was refreshed to exclude previous treatment subjects, and cells were cultured for another 24 h. Subsequently, the resulting medium was collected to treat the activated mouse T cells, which were isolated from spleen and stimulated with anti-CD28 and anti-CD3. (B) GPR43 expression in MCoEpiCs by flow cytometry. n = 4. (C) The resulting Medium was collected to determine IFN-γ concentration. n = 4. (D) Treated CD4^+^ T cells were analyzed for Th1 markers and the ratio of Th1 cells out of CD4^+^ T cells was calculated. n = 4. (E) Experimental scheme for (F and G). Naïve CD4^+^ T cells were isolated from mouse spleen and activated with anti-CD3, anti-CD28, and Th1 polarization proteins, then treated with GLPG. Supernatant from fermentations was used to treat the resulting T cells above, followed by 0.5 % (w/v) DSS treatment. (F and G) Th1, Th2, Th17, and Treg markers were determined both in mRNA and protein levels by qRT-PCR and flow cytometer to detect the effect of the fermentation on Th1 functions including cytokine secretions and differentiation potentials to other effector T cells. Data are representative from one of two independent experiments, n = 4. Data are presented as mean ± SEM. Statistical significance was determined using one-way ANOVA, followed by Tukey's multiple-comparison tests or Student's t-test. *P ≤ 0.05, **P ≤ 0.01. See also Fig. S6.

**Fig. 6:**
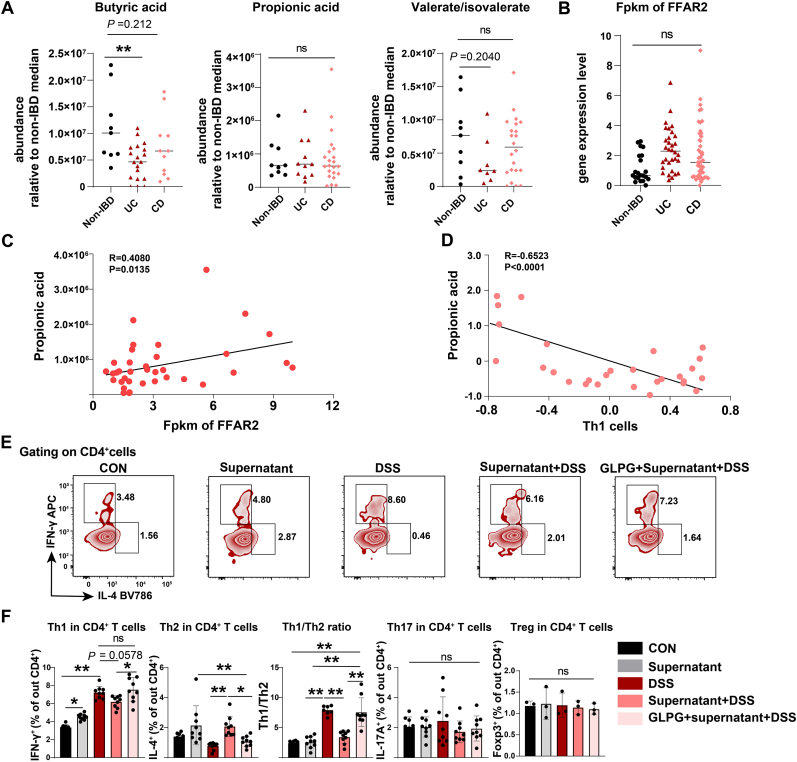
**EGCG fermentation inhibits Th1 differentiation via colonic GPR43 in human.** Data resources for (A–D). SCFA concentrations in human stools (including non-IBD, Crohn's disease (CD), and Ulcerative colitis (UC) patients) were shown. Untargeted metabolic data were obtained from Project PR000639 in the Metabolomics Workbench (http://www.metabolomicsworkbench.org). (A) Concentrations of SCFA were from peak area and shown as abundance relative to non-IBD. Data are shown in median. n = 9–22. (B) Bulk RNA-sequencing data for biopsies collected 10 cm from rectum are is accessed through GEO Series accession number GSE111889. *FFAR2* expressions among non-IBD, UC, and CD patients were shown. n = 21–48. (C) Correlation of *FFAR2* gene expression with propionic acid abundance in corresponding UC and CD patients was shown (r = 0.4080, p = 0.0135; Two-tailed Pearson correlation coefficient; Linear regression with 95 % confidence). Th1-related genes in UC and CD patients were scored based on gene set variation analysis (GSVA) enrichment score. The resulting scores were shown. (D) Correlation between the resulting scores for Th1-related genes and propionic acid abundance in corresponding UC and CD patients were analyzed (r = −0.6523, p < 0.0001; Two-tailed Pearson correlation coefficient; Linear regression with 95 % confidence). n = 30. (E and F) EGCG and colon contents were fermented, and the fermentations was centrifuged, then concentrated at low temperature and diluted with cell culture medium. The resulting supernatant was used to treat human colonic epithelial cells (HCoEpiCs) for 24 h after HCoEpiCs were treated with GLPG. Then culture medium was refreshed and followed by 2.5 % (w/v) DSS treatment. The culture medium was refreshed and epithelial cells were cultured for another 24 h. Subsequently, the above Medium was collected to treat the activated human CD4^+^ T cells, which were isolated from PBMC and stimulated with PHA-L. Th1, Th2, Th17, and Treg markers were determined by flow cytometer after cells were stained. Data are representative from one of two independent experiments, n = 3–9. Data are presented as mean ± SEM. Statistical significance was determined using one-way ANOVA, followed by Tukey's multiple-comparison tests or Student's t-test. *P ≤ 0.05, **P ≤ 0.01. See also Fig. S7.

We then explored the molecular mechanisms by which EGCG augments GPR43 activity. We performed dynamic docking analysis to assess the binding affinities of EGCG, acetate, and propionate with the GPR43 receptor. As expected, both acetate and propionate exhibited high binding affinities with GPR43 (Fig. 4I). We also identified a set of binding sites (i.e., R180, H242, R255, Y238, Y94, Y90, and V144) within a hydrophobic pocket responsible for the direction interaction of acetate and propionate with GPR43. In contrast, EGCG exhibited no interaction with the GPR43 protein, as its binding energy greater than 0 kcal/mol (binding energy is +383.22), suggesting a weak or unfavorable binding interaction with a low probability of molecular binding (data not shown). Given the lack of a direct effect of EGCG on Th1 cells *in vitro*, the absence of a direct EGCG-GPR43 interaction may account for the insignificant direct effect of EGCG on CD4^+^ effector Th1 cells and GPR43 signaling cascades. Indeed, no significant change in GPR43 protein expression on naïve CD4^+^ T cells was observed upon direct treatment by EGCG *in vitro* (Fig. S5D). Overall, the above results suggest that EGCG-mediated increases in intestinal GPR43 activation involve in *Ffar2* transcription but not by a direct EGCG-GPR43 interaction.

### 3.4 Microbiome-reshaping inhibits Th1 polarization in an intestinal epithelial GPR43-dependent manner

Given that GPR43 is highly expressed in IECs and our docking analysis suggests a high affinity between acetate/propionate and GPR43, we therefore sought to determine whether GPR43 is required for microbes to mediate the suppressive effects of EGCG on DSS-induced Th1 cell polarization. First, we established an *in vitro* mouse colonic epithelial cell and primary CD4^+^ naïve T cell culture model. The fermented supernatant of colon feces and EGCG were used to incubate colonic epithelial cells for 24 h after using GLPG to inhibit GPR43 expression, followed by DSS treatment to mimic inflammatory conditions *in vivo*. The supernatant was subsequently transferred to activated T cells isolated from mouse spleen and stimulated with anti-CD28 and anti-CD3 (Fig. 5A). Consistent with our *in vivo* findings, the EGCG-fermented fecal supernatant exhibited increased acetate and propionate concentrations (Fig. S6A). Correspondingly, the EGCG-fermented supernatant increased GPR43 expression in mouse colonic epithelial cells (MCoEpiCs) (Fig. 5B) and reversed the DSS-induced up-regulation of IFN-γ, but not IL-12 (Fig. 5C and S6B), accompanied by an inhibition of gene expression of the three NF-κB subunits (Fig. S6C). Meanwhile, the inhibition of Th1 polarization by the EGCG-fermented supernatant was rescued by the addition of GLPG preintervention in DSS-treated MCoEpiCs (Fig. 5D and S6C). Similar to the *in vivo* findings, the other effector T cell populations (including Th2, Th17, and Treg) remained unchanged by EGCG fermentation with GLPG treatment, compared to the fermentation-treated DSS group (Fig. S6D).

Furthermore, we examined the direct impact of EGCG fermentation on Th1 cell functions and the *trans*-differentiation potential of Th1 cells to other types of T cells. Mouse naïve T cells were isolated, activated, and polarized to Th1 cells, followed by the GLPG plus EGCG fermentation treatment. Cells were then stimulated with 0.5 % DSS for 24 h (Fig. 5E). The qRT-PCR results showed that the expression levels of Th1 cell-associated cytokines and transcription factors were not changed, except for IFN-γ (Fig. 5F), suggesting that EGCG fermentation potentially influences IFN-γ-related cell functions in Th1 cells. Further, flow cytometry results revealed no changes in the representative mouse T cell markers IL-10, IL-4, IL-17A, and Foxp3 upon EGCG fermentation treatment, indicating that EGCG fermentation did not affect the differentiation potentials of Th1 cell to other effector T cells (Fig. 5G). Therefore, the above results suggest that an interaction of intestinal microecology with host epithelial GPR43 is required for EGCG's protective effects against pathogenic Th1 cell polarization in the context of DSS-induced colitis.

### 3.5 EGCG fermentation inhibits Th1 polarization via colonic GPR43 in human

To further explore the clinical implication of key components of this EGCG-regulated multilevel intestinal microecosystem, we first analyzed the enrichment of fecal SCFAs, including propionic acid, butyric acid, and valerate/isovalerate, and the gene expression of colon epithelial *FFAR2* in primary patients with Crohn's disease (CD) or Ulcerative colitis (UC) using a public database (untargeted metabolic data from Project ID PR000639 in the Metabolomics Workbench (http://www.metabolomicsworkbench.org); bulk RNA-seq data are from GSE111889) [24]. There were no significant differences in the levels of propionic acid and *FFAR2* in the primary biopsies between non-IBD patients and IBD patient (UC, CD) groups, except for a down-regulation of butyric acid in UC and CD, and valerate/isovalerate in UC samples, suggesting an inactive state for these microenvironmental components in primary samples (Fig. 6A and B). Further expression correlation analysis revealed a significant positive correlation between fecal propionic acid and colonic *FFAR2* levels (Fig. 6C), and a significant negative correlation between fecal propionic acid enrichment and colonic Th1 cell scores (Th1-related genes in UC and CD patients were scored based on gene set variation analysis (GSVA) enrichment score) (Fig. 6D).

Additionally, we explored the effects of EGCG fermentation and colonic GPR43 on human Th1 cell polarization and functions via *in vitro* induction and microenvironment co-culture models of primary CD4^+^ naïve T cells isolated from human PBMC (Figs. S7A and S7B). Flow cytometry results showed that EGCG fermentation supernatant reversed DSS-induced upregulation expression of IFN-γ, and downregulation of Th2 cell marker IL-4, but not IL-17A or Foxp3 (Fig. 6E and F). This was also accompanied by a decrease in gene levels of *NFKB1* and *NFKB2* in epithelial cells and a downregulation of IFN-γ level in the cell culture medium (Figs. S7C and S7D). These effects could be rescued by an addition of GLPG preintervention in DSS-treated HCoEpiCs (Fig. 6E and F). In addition, we observed that the EGCG fermentation supernatant increased GPR43 expression in HCoEpiCs (Fig. S7E). Altogether, these data suggest that this EGCG-regulated multilevel-regulated intestinal microecosystem has clinical implications for humans.

## 4 Discussion

Currently, the conventional pharmacological treatments lack long-lasting efficacy due to the rapid emergence of drug resistance or off-target effects [33,34]. Reconstruction of intestinal microecology, such as targeting gut microbes or microbial pathways, emerges as a promising therapeutic strategy for IBD treatment. EGCG, as a promising natural compound with low toxicity, displays various therapeutic effects, and is undergoing clinical trials (from Phase 1–4) in many diseases, including pulmonary fibrosis [35], Alzheimer's disease [36], and COVID-19 [37]. EGCG also shows strong anti-inflammatory effects in IBD by regulating some major immune cell populations, individual microbiota, and cytokine release [17]. However, the following key scientific issues still remain unclear: (1) Further responding immune subsets of EGCG; (2) What the impact of EGCG on the interplay between key intestinal microenvironment compositions involving immune cells, microbiome, epithelial cells, metabolites, and immune molecular effectors; (3) What the discrepancy on the therapeutic effects of EGCG between with and without supports of intestinal microenvironment. Clarification of these issues doubtlessly contributes to the development of novel EGCG-based immunotherapy strategies and related clinical trials.

To uncover the responding immune cell subsets of EGCG in IBD, flow cytometry analysis of canonical T cell compartment, CD79a^+^ B cell, and macrophage was performed in colitis mice. We find that EGCG pretreatment significantly suppresses the number of CD4^+^ T cells and increases macrophages but it does not influence B cell or CD8αβ^+^ T cell populations. Through RNA-seq analysis of four main intestinal effector T cell populations, we observe that marker genes of Th1, Th17, and Treg cells are susceptible (inhibition) to EGCG treatment, although Th2 cells are recovered to some extent. In our study, Th1 cells are the most affected subsets, and EGCG markedly affects effector CD4^+^ T cell populations under the colitis condition, especially Th1 cells. Previous studies show that EGCG inhibits major CD3^+^ T cell infiltration in inflammatory colonic tissues [38]. Yang et al. reported EGCG pretreatment significantly increases the number of macrophages in the abdominal cavity and peripheral blood of mice [39]. Interestingly, CD4^+^ T cells are considered a key interactive cell population of macrophages in IBD [40]. For example, some studies demonstrate that intestinal macrophages maintain T cell function by scavenging dead cells and remodeling epithelial cells [41]. In addition, intestinal macrophages in patients with IBD induce Th1 and Th17 polarization, seemingly caused by the accumulation of immature macrophages [[42], [43], [44]]. Therefore, it is possible that the up-regulation of macrophages potentially enhance the inhibitory efficacy of EGCG through activating more naïve CD4^+^ T cells and facilitating their targeting by EGCG.

We further explore whether intestinal microenvironment mediates the impact of EGCG on effector T cells. Using an *in vitro* induction and culture model of primary activated T cells/Th1 cells, we find that neither activated T cells without polarization nor Th1 cells are influenced by EGCG under the colitis condition, as reflected by no significant alteration in the expression levels of some key cell function-related factors and other T lineages’ makers of activated T and polarized Th1 cells. Therefore, it may indicate a poor efficiency for active T cells to directly absorb EGCG, potentially due to a lack of sufficiently expressed binding molecules at plasma membranes or other physicochemical characteristics, such as lipid solubility and quantity of electricity. Through *in vivo* colitis mouse model supplemented with an antibiotic cocktail, we confirm intestinal microbes are essential for the protective effects of EGCG on colitis progression and its inhibitory effects on the Th1 subset. Particularly, elimination of microbes alone has subtle influences on the phenotype of colitis, indicating that the remodeling of microflora and microenvironmental metabolites caused by EGCG pretreatment is critical for its biological effects. Further, 16S rRNA sequencing analysis reveals a series of novel intestinal probiotic strains are enriched with EGCG treatment in either normal or colitis conditions. Specifically, in normal condition, probiotic strains *Lachnoclostridium*, *Lactobacillus*, *Candidatus-Saccharimonas*, *Alloprevotella*, *Leuconostoc*, and *Weissella* are enriched, while in colitis condition, *Ruminococcus*, *Dubosiella*, *Akkermansia*, *Bacteroides*, *Eubacterium*, *Romboutsia*, and *Enterococcus* are enriched. These bacteria possess key traits that potentially contribute to protecting of intestinal integrity and environments: (1) Producing SCFAs for anti-inflammatory and immune-suppressive effects, effectively stabilizing the gut environment, such as *Lachnoclostridium, Akkermansia*, *Ruminococcus, Bacteroides, Eubacterium*, and *Romboutsia* [33,45]; (2) Producing natural antibiotics that inhibit pathogenic bacteria, such as *Enterococcus* [46]; (3) Maintaining a balanced intestinal microbiota, such as *Lactobacillus* [47]; (4) Maintaining gut acid-base balance via carbohydrate metabolism, such as *Candidatus-Saccharimonas* [48]. Notably, many pathogenic bacteria that promote inflammation are significantly inhibited upon EGCG treatment under the colitis condition, including *Erysipelatoclosstridium*, *Helicobacter*, *Prevotellaceae*, *Escherichia-Shigella*, and *Oscillibacter*. High throughput metabolomics confirm that two SCFAs (i.e. acetic acid and propionic acid) are dramatically increased under the same condition. In particular, we confirm a strong negative correlation between the concentration of SCFAs in feces and the GSVA scores of intestinal Th1 cells in human IBD clinical samples. These results indicate that reshaping gut microbiota, especially enriching SCFA-producing bacteria, is indispensable for the optimal functioning of EGCG in the intestinal microenvironment.

Intestinal epithelial cells are an integral component of the intestinal microenvironment. However, whether it is involved in regulating the effects of EGCG-driven microbiota reshaping on intestinal immune cells remains unknown. Using *in vitro* induction and culture model of primary Th1 cells, we find no discrepancy in Th1 cell numbers and *trans*-differentiation potential between Th1 cells with and without treatment by the supernatant from EGCG fermentation under the colitis condition. Importantly, when primary effector T cells were incubated by the medium that cultured colon epithelial cells stimulated by EGCG fermentation, the number of Th1 cells is significantly suppressed, accompanied by the down-regulation of secreted IFN-γ level. Therefore, our results suggest intestinal epithelial cells as well as IFN-γ are also essential for protective effects of EGCG in intestinal microenvironment. Given that GPR43 interacts with acetate and propionate the most among SCFAs, we speculate GPR43 may be a key immune molecular effector for EGCG. Particularly, our immunofluorescence results show no significant increase in the expression levels of GPR43 in Th1 cells and IEC upon EGCG treatment *in vitro* without the microenvironment. RNA-seq data also reveal that typical down-stream pathways of GPR43 are not altered in colon epithelial cells. Moreover, our molecular docking analysis demonstrates a low possibility for a direct interaction between EGCG and GPR43. Therefore, a low expression of GPR43 in Th1 cells and an absence of EGCG-GPR43 interaction may explain why acetate/propionate and EGCG do not directly affect T cell activity. Using the *in vivo* colitis model and *in vitro* induction and microenvironment co-culture models of primary effector T cells from mouse and human, we find that EGCG displays an enhanced effects on expression levels of colonic GPR43 and its activity. Correspondingly, the GPR43 inhibitor intercepts the effects of EGCG on colonic GPR43 and Th1 cells. These results indicate that colonic GPR43 is key effector molecule for EGCG functions in the colitis microenvironment. To date, the binding pocket of GPR43 to acetate/propionate remain unknown. Through molecular docking analysis, a hydrophobic pocket mainly consists of R180, H242, R255, Y238, Y94, Y90, and V144 is responsible for its binding to the acetate/propionate. This binding pocket involves transmembrane helices III, IV, and VI as well as two extracellular topological domains. Although hydrophobic tail bases of acetate/propionate may form hydrophobic contacts with this pocket, alternatively, the carboxyl group of acetate/propionate may contribute to a network of interactions with the pocket through H-bonding, their specific binding mechanisms still need further elucidation.

**Fig. 7:**
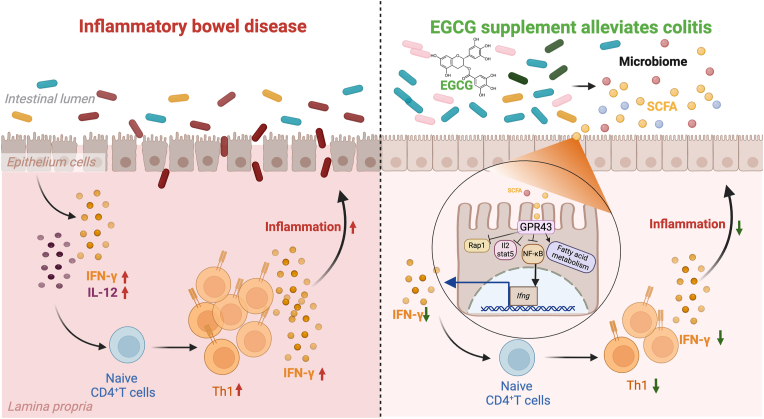
**EGCG inhibits Th1 cell polarization and self-amplification in a manner that required multilevel regulated intestinal microecosystem**. In the colitis condition, pathogenic bacteria dominated in the intestinal lumen damage the epithelial lining, causing increased permeability, which allows damaged epithelial cells and activated immune cells to produce pro-inflammatory cytokines, including IFN-γ and IL-12, leading to a promotion of Th1 cell polarization and self-amplification, and contributing to the chronic inflammation in IBD. Upon EGCG treatment, a variety of probiotics and environmental acetic acid/propionic acid are enriched, and a direct binding of acetic acid/propionic acid to the epithelial GPR43 activates down-stream pathways, and subsequently lead to a decrease in IFN-γ release, which promotes the repair of damaged epithelial cells through reducing Th1 cell polarization and numbers.

Collectively, our study uncovers a novel interplay between EGCG and colitis microenvironment involved in Th1 cells, gut microbiota remodeling, SCFAs, intestinal epithelial cells, and colonic GPR43 in mouse models and human samples. This interplay determines the optimal functions of EGCG to inhibit Th1 polarization and self-amplification, eventually ameliorating colitis symptoms (Fig. 7). Our study also highlights a new theoretical foundation that combination intervention targeting key components of this multistage regulation system can be a promising drug combination strategy for EGCG.

## Data, materials, and softeare availability

All data necessary to evaluate the conclusions of the paper can be found in the paper and/or the Supplementary Materials. Additional data related to this paper may be requested from the authors. RNA-seq and 16S rDNA sequencing data generated in this paper are available in National Genomics Data Center under the accession number OMIX005839 and OMIX005840.

## CRediT authorship contribution statement

**Siyan Che:** Writing – review & editing, Writing – original draft, Resources, Methodology, Investigation, Conceptualization. **Beibei Qin:** Visualization, Software, Methodology, Formal analysis, Data curation. **Kunfu Wu:** Software, Methodology, Formal analysis, Data curation. **Mingzhi Zhu:** Methodology, Data curation. **Han Hu:** Methodology, Investigation. **Can Peng:** Methodology, Investigation. **Zi Wang:** Writing – review & editing, Resources, Methodology, Data curation, Conceptualization. **Yulong Yin:** Supervision, Project administration. **Yaoyao Xia:** Writing – review & editing, Resources, Methodology, Data curation, Conceptualization. **Miaomiao Wu:** Writing – review & editing, Writing – original draft, Resources, Methodology, Project administration, Investigation, Funding acquisition, Data curation, Conceptualization.

## Declaration of competing interest

The authors declare that they have no known competing financial interests or personal relationships that could have appeared to influence the work reported in this paper.

## Data Availability

Data will be made available on request.

## References

[1] Jaeger N., Gamini R., Cella M. (2021). Single-cell analyses of Crohn's disease tissues reveal intestinal intraepithelial T cells heterogeneity and altered subset distributions. Nat. Commun..

[2] Du Y., Ding H., Vanarsa K. (2019). Low dose epigallocatechin gallate alleviates experimental colitis by subduing inflammatory cells and cytokines, and improving intestinal permeability. Nutrients.

[3] Pan X., Zhu Q., Pan L.L., Sun J. (2022). Macrophage immunometabolism in inflammatory bowel diseases: from pathogenesis to therapy. Pharmacol. Therapeut..

[4] Xie M., Zhu Y., Zhou Y. (2023). Interleukin-35 -producing B cells rescues inflammatory bowel disease in a mouse model via STAT3 phosphorylation and intestinal microbiota modification. Cell Death Dis..

[5] Shan Y., Lee M., Chang E.B. (2022). The gut microbiome and inflammatory bowel diseases. Annu. Rev. Med..

[6] de Souza H.S., Fiocchi C. (2016). Immunopathogenesis of IBD: current state of the art. Nat. Rev. Gastroenterol. Hepatol..

[7] Plichta D.R., Graham D.B., Subramanian S., Xavier R.J. (2019). Therapeutic opportunities in inflammatory bowel disease: mechanistic dissection of host-microbiome relationships. Cell.

[8] Neurath M.F. (2019). Targeting immune cell circuits and trafficking in inflammatory bowel disease. Nat. Immunol..

[9] Fan L., Xia Y., Wang Y. (2023). Gut microbiota bridges dietary nutrients and host immunity. Sci. China Life Sci..

[10] Yilmaz B., Juillerat P., Øyås O. (2019). Microbial network disturbances in relapsing refractory Crohn's disease. Nat. Med..

[11] Tillack C., Ehmann L.M., Friedrich M. (2014). Anti-TNF antibody-induced psoriasiform skin lesions in patients with inflammatory bowel disease are characterised by interferon-γ-expressing Th1 cells and IL-17A/IL-22-expressing Th17 cells and respond to anti-IL-12/IL-23 antibody treatment. Gut.

[12] Shirakami Y., Shimizu M., Tsurumi H., Hara Y., Tanaka T., Moriwaki H.E.G.C.G., Polyphenon E. (2008). Attenuate inflammation-related mouse colon carcinogenesis induced by AOM plus DDS. Mol. Med. Rep..

[13] Liu S., Cao Y., Ma L. (2022). Oral antimicrobial peptide-EGCG nanomedicines for synergistic treatment of ulcerative colitis. J. Contr. Release : Off. J. Controll. Release Soc..

[14] Mehta M., Ahmed S., Dryden G. (2018). Refractory pouchitis improves after administration of the green tea polyphenol EGCG: a retrospective review. Int. J. Colorectal Dis..

[15] Jang J.Y., Lee J.K., Jeon Y.K., Kim C.W. (2013). Exosome derived from epigallocatechin gallate treated breast cancer cells suppresses tumor growth by inhibiting tumor-associated macrophage infiltration and M2 polarization. BMC Cancer.

[16] Park S., Jeong G.H., Jee S.H., Kim T.H., Kim S.B. (2022). Mechanism of (-)-epigallocatechin gallate (EGCG) dimerization by low-temperature plasma. Sci. Rep..

[17] Cai F., Liu S., Lei Y. (2021). Epigallocatechin-3 gallate regulates macrophage subtypes and immunometabolism to ameliorate experimental autoimmune encephalomyelitis. Cell. Immunol..

[18] Liu Z., de Bruijn W.J.C., Bruins M.E., Vincken J.P. (2020). Reciprocal interactions between epigallocatechin-3-gallate (EGCG) and human gut microbiota in vitro. J. Agric. Food Chem..

[19] Reikvam D.H., Erofeev A., Sandvik A. (2011). Depletion of murine intestinal microbiota: effects on gut mucosa and epithelial gene expression. PLoS One.

[20] Yang L., Lin H., Lin W., Xu X. (2020). Exercise ameliorates insulin resistance of type 2 diabetes through motivating short-chain fatty acid-mediated skeletal muscle cell autophagy. Biology.

[21] Aggarwal N., Deerhake M.E., DiPalma D. (2021). Secreted osteopontin from CD4(+) T cells limits acute graft-versus-host disease. Cell Rep..

[22] Teratani T., Mikami Y., Nakamoto N. (2020). The liver-brain-gut neural arc maintains the T(reg) cell niche in the gut. Nature.

[23] Stoddart L.A., Smith N.J., Jenkins L., Brown A.J., Milligan G. (2008). Conserved polar residues in transmembrane domains V, VI, and VII of free fatty acid receptor 2 and free fatty acid receptor 3 are required for the binding and function of short chain fatty acids. J. Biol. Chem..

[24] Wang Z., Wang P., Zhang J. (2023). The novel GATA1-interacting protein HES6 is an essential transcriptional cofactor for human erythropoiesis. Nucleic Acids Res..

[25] Lloyd-Price J., Arze C., Ananthakrishnan A.N. (2019). Multi-omics of the gut microbial ecosystem in inflammatory bowel diseases. Nature.

[26] Younes M., Aggett P., Aguilar F. (2018). Scientific opinion on the safety of green tea catechins. EFSA J. Euro. Food Saf. Auth..

[27] Nair A.B., Jacob S. (2016). A simple practice guide for dose conversion between animals and human. J. Basic Clin. Pharm..

[28] Hu Y., Gu J., Lin J. (2021). (-)-Epigallocatechin-3-gallate (EGCG) modulates polarized macrophages to suppress M1 phenotype and promote M2 polarization in vitro and in vivo. J. Funct.Foods.

[29] Danese S., Vermeire S., Hellstern P. (2019). Randomised trial and open-label extension study of an anti-interleukin-6 antibody in Crohn's disease (ANDANTE I and II). Gut.

[30] Kennedy N.A., Heap G.A., Green H.D. (2019). Predictors of anti-TNF treatment failure in anti-TNF-naive patients with active luminal Crohn's disease: a prospective, multicentre, cohort study. Lancet Gastroenterol. Hepatol..

[31] Deleu S., Machiels K., Raes J., Verbeke K., Vermeire S. (2021). Short chain fatty acids and its producing organisms: an overlooked therapy for IBD?. EBioMedicine.

[32] Morrison D.J., Preston T. (2016). Formation of short chain fatty acids by the gut microbiota and their impact on human metabolism. Gut Microb..

[33] Zheng H., Xu P., Jiang Q. (2021). Depletion of acetate-producing bacteria from the gut microbiota facilitates cognitive impairment through the gut-brain neural mechanism in diabetic mice. Microbiome.

[34] Feng W., Ao H., Peng C. (2018). Gut microbiota, short-chain fatty acids, and herbal medicines. Front. Pharmacol..

[35] Donà M., Dell'Aica I., Calabrese F. (2003). Neutrophil restraint by green tea: inhibition of inflammation, associated angiogenesis, and pulmonary fibrosis. J. Immunol..

[36] Bieschke J., Russ J., Friedrich R.P. (2010). EGCG remodels mature alpha-synuclein and amyloid-beta fibrils and reduces cellular toxicity. Proc. Natl. Acad. Sci. U.S.A..

[37] Xu H., Li S., Liu J. (2023). Bioactive compounds from Huashi Baidu decoction possess both antiviral and anti-inflammatory effects against COVID-19. Proc. Natl. Acad. Sci. U.S.A..

[38] Zhang S., Liu X., Mei L., Wang H., Fang F. (2016). Epigallocatechin-3-gallate (EGCG) inhibits imiquimod-induced psoriasis-like inflammation of BALB/c mice. BMC Compl. Alternative Med..

[39] Yang Y., Han X., Chen Y. (2021). EGCG induces pro-inflammatory response in macrophages to prevent bacterial infection through the 67LR/p38/JNK signaling pathway. J. Agric. Food Chem..

[40] Kondo A., Ma S., Lee M.Y.Y. (2021). Highly multiplexed image analysis of intestinal tissue sections in patients with inflammatory bowel disease. Gastroenterology.

[41] Zigmond E., Varol C., Farache J. (2012). Ly6C hi monocytes in the inflamed colon give rise to proinflammatory effector cells and migratory antigen-presenting cells. Immunity.

[42] Kamada N., Hisamatsu T., Honda H. (2009). Human CD14+ macrophages in intestinal lamina propria exhibit potent antigen-presenting ability. J. Immunol..

[43] Ogino T., Nishimura J., Barman S. (2013). Increased Th17-inducing activity of CD14+ CD163 low myeloid cells in intestinal lamina propria of patients with Crohn's disease. Gastroenterology.

[44] Han X., Ding S., Jiang H., Liu G. (2021). Roles of macrophages in the development and treatment of gut inflammation. Front. Cell Dev. Biol..

[45] Olsson L.M., Boulund F., Nilsson S. (2022). Dynamics of the normal gut microbiota: a longitudinal one-year population study in Sweden. Cell Host Microbe.

[46] Hanchi H., Mottawea W., Sebei K., Hammami R. (2018). The genus Enterococcus: between probiotic potential and safety concerns-an update. Front. Microbiol..

[47] von Schillde M.A., Hörmannsperger G., Weiher M. (2012). Lactocepin secreted by Lactobacillus exerts anti-inflammatory effects by selectively degrading proinflammatory chemokines. Cell Host Microbe.

[48] Ding T., Xu M., Li Y. (2022). An overlooked prebiotic: beneficial effect of dietary nucleotide supplementation on gut microbiota and metabolites in senescence-accelerated mouse prone-8 mice. Front. Nutr..

