# Supplementary material for: EGCG drives gut microbial remodeling-induced epithelial GPR43 activation to lessen Th1 polarization in colitis
Source: Redox Biol. 2024 Jul 30;75:103291. doi: 10.1016/j.redox.2024.103291 (PMC11363845; doi:10.1016/j.redox.2024.103291)
Supplement: Multimedia component 1 [file mmc1.docx]

**Supplementary Materials**
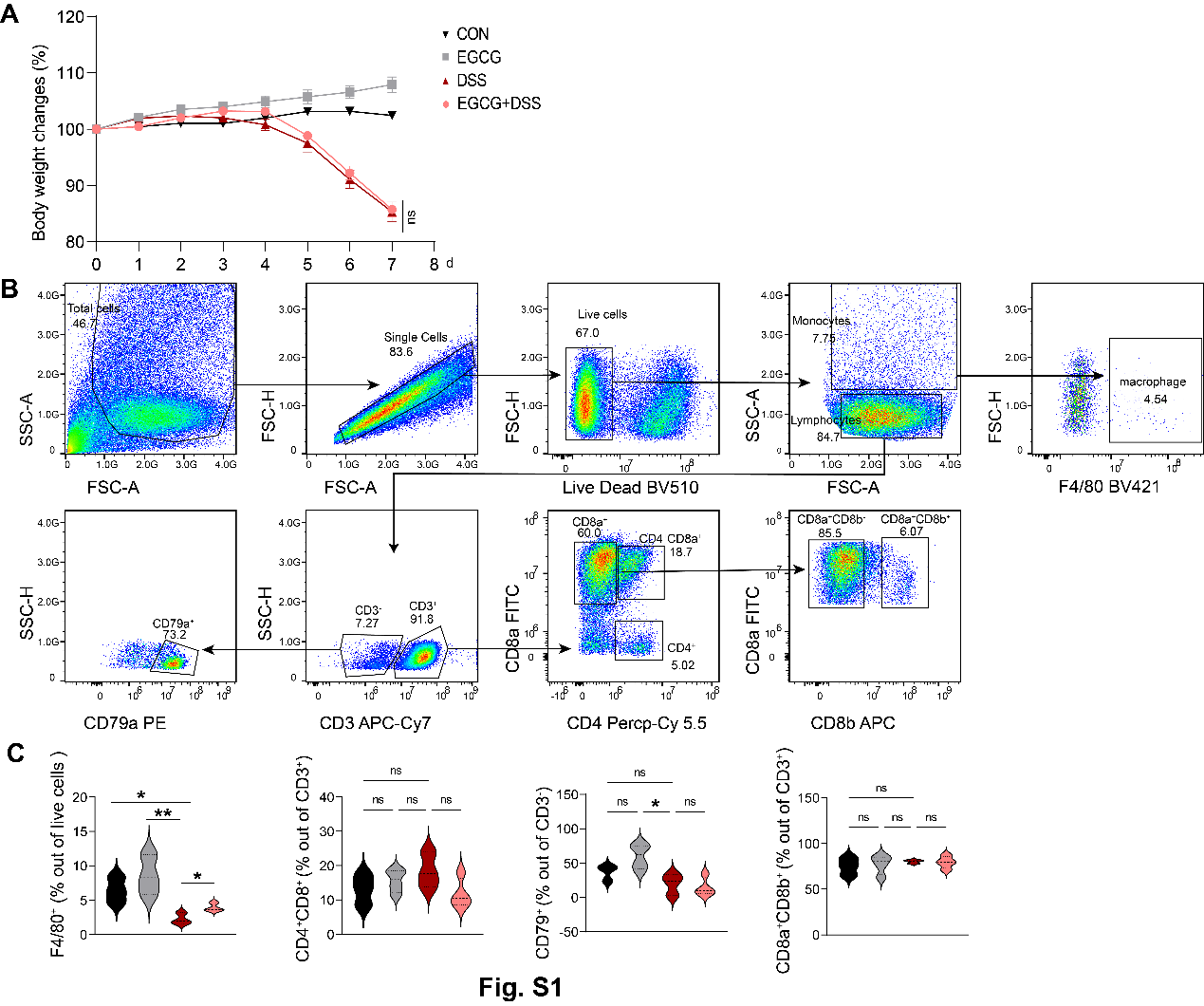


**Figure S1, related to Figure 1. Effects of EGCG on intestinal immune cells in colitis mice.**

Mice were administrated 2.5% (w/v) DSS in drinking water for 5 days after they were treated with 80 mg/kg/day EGCG via oral gavage for 21 days, then allowed to recover for 2 days using normal drinking water.

(A) Body weight was recorded daily to calculate body weight change. n = 8-12.

(B) Colonic T cell subtypes were detected by flow cytometry after colon immune cells were isolated, and gating strategy was shown.

(C) Major immune cells types were analyzed and quantified by flow cytometry. Two to three mice were pooled for one sample, data are one representative of two independent experiments. n = 3.

Data are presented as mean ± SEM. Statistical significance was determined using one-way ANOVA, followed by Tukey’s multiple-comparison tests or Student’s t-test. * *P* ≤ 0.05, ** *P* ≤ 0.01.


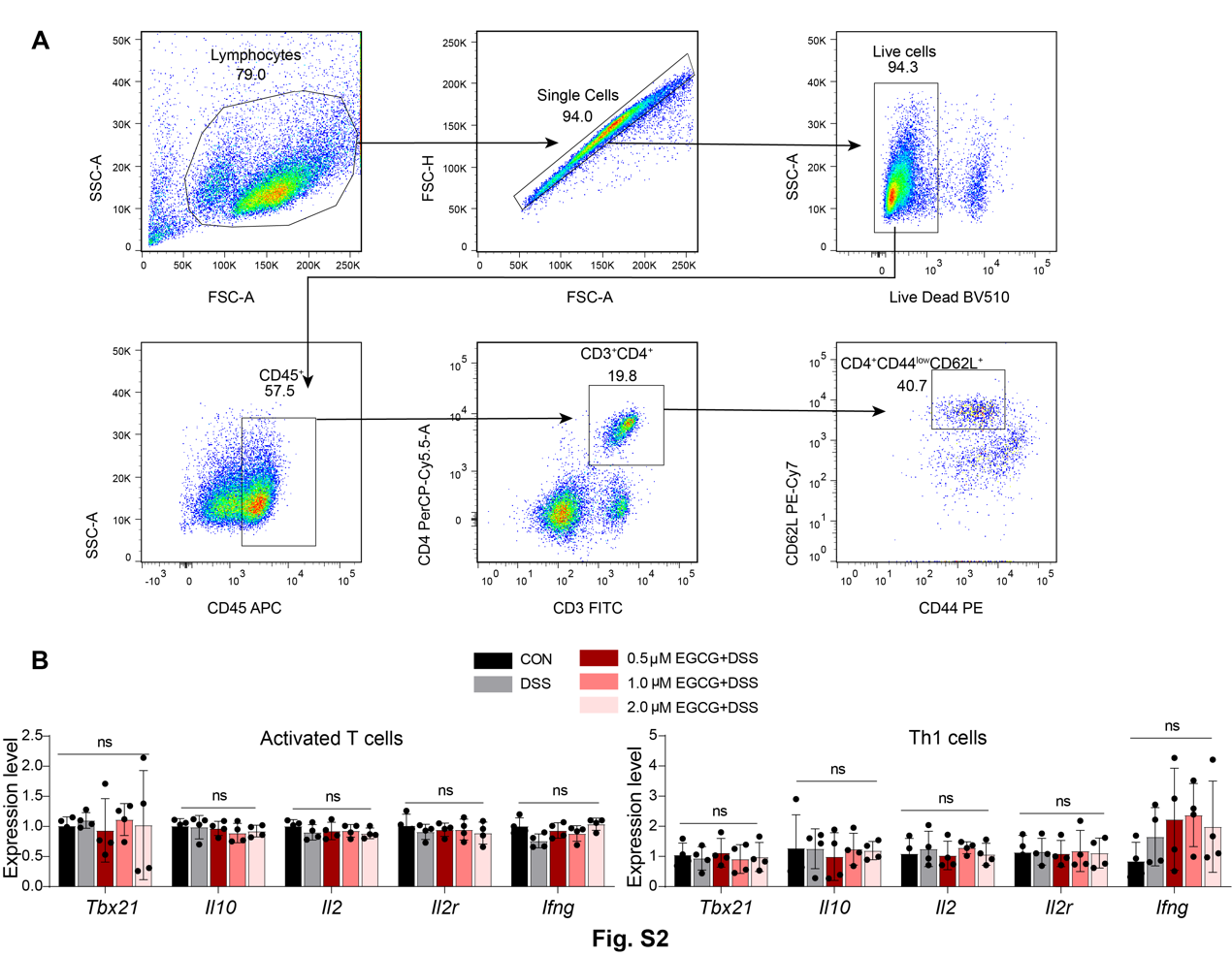


**Figure S2, related to Figure 2. EGCG cannot directly regulate mouse activated T or Th1 cells functions in DSS-induced inflammatory condition.**

Naïve CD4^+^ T cells from mouse spleens were isolated, activated in vitro using anti-CD28 and anti-CD3, and induced Th1 polarization. Cells were then stimulated with 0.5% (w/v) DSS after incubation of EGCG with different concentrations (0.5 μM, 1.0 μM, and 2.0 μM) for 24 hours.

(A) Gating strategy for sorting naïve CD4^+^ T cells from mouse spleen was shown.

(B) Cellular functional factors were detected at the mRNA level for activated T cells and Th1 cells treated with or without EGCG. Data are one representative of two independent experiments. n = 4.

Data are presented as mean ± SEM. Statistical significance was determined using unpaired Student’s t-test. * *P* ≤ 0.05, ** *P* ≤ 0.01.


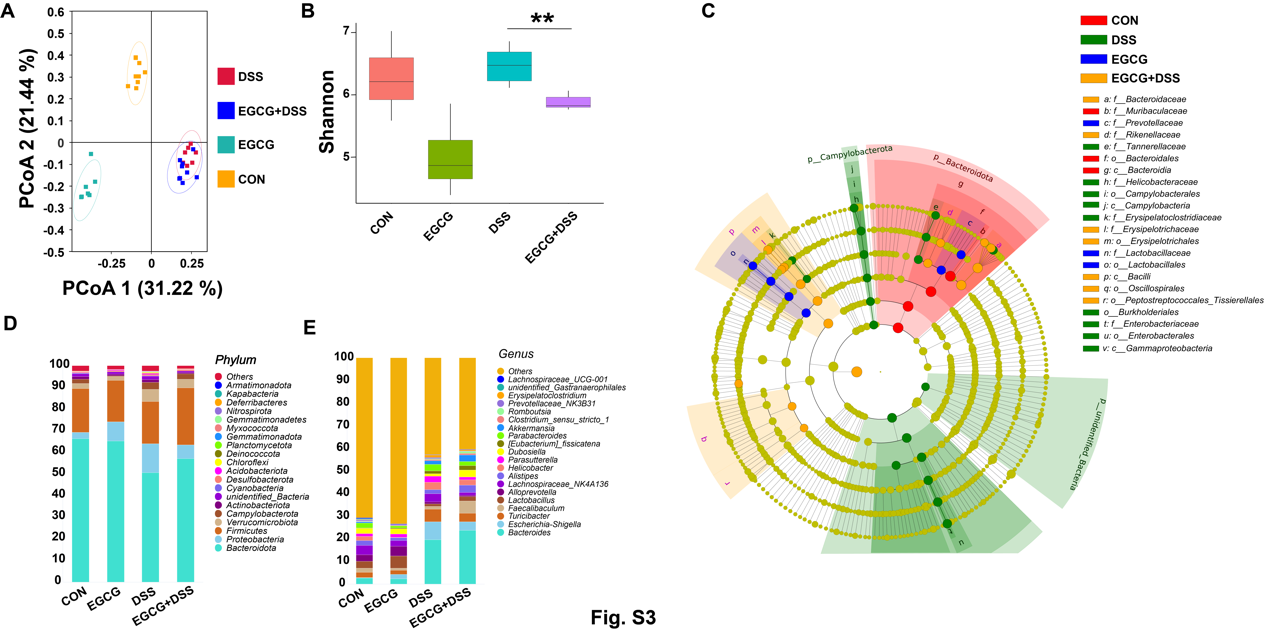
 **Figure S3, related to Figure 2.** EGCG reshapes gut microbes in colitis mice.

Mice were orally gavaged with 80 mg/kg/day EGCG for 21 days, then given drinking water containing 2.5%(w/v) DSS for 5 days, followed by 2 days of recovery. 16S rDNA sequencing for colon feces was performed via the 454 FLXtitanium system and sequences were analyzed using QIIME software.

(A and B) PCoA analysis (A) and Shannon index (B). n = 8-10. Data are presented as mean ± SEM. Statistical significance was determined using unpaired Student’s t-test. * *P* ≤ 0.05, ** *P* ≤ 0.01.

(C) Microbiota compositions from different groups were shown by Cladogram. n = 8-10.

(D and E) Bacterial difference at the phylum (D) and genus (E) levels. n = 8-10.


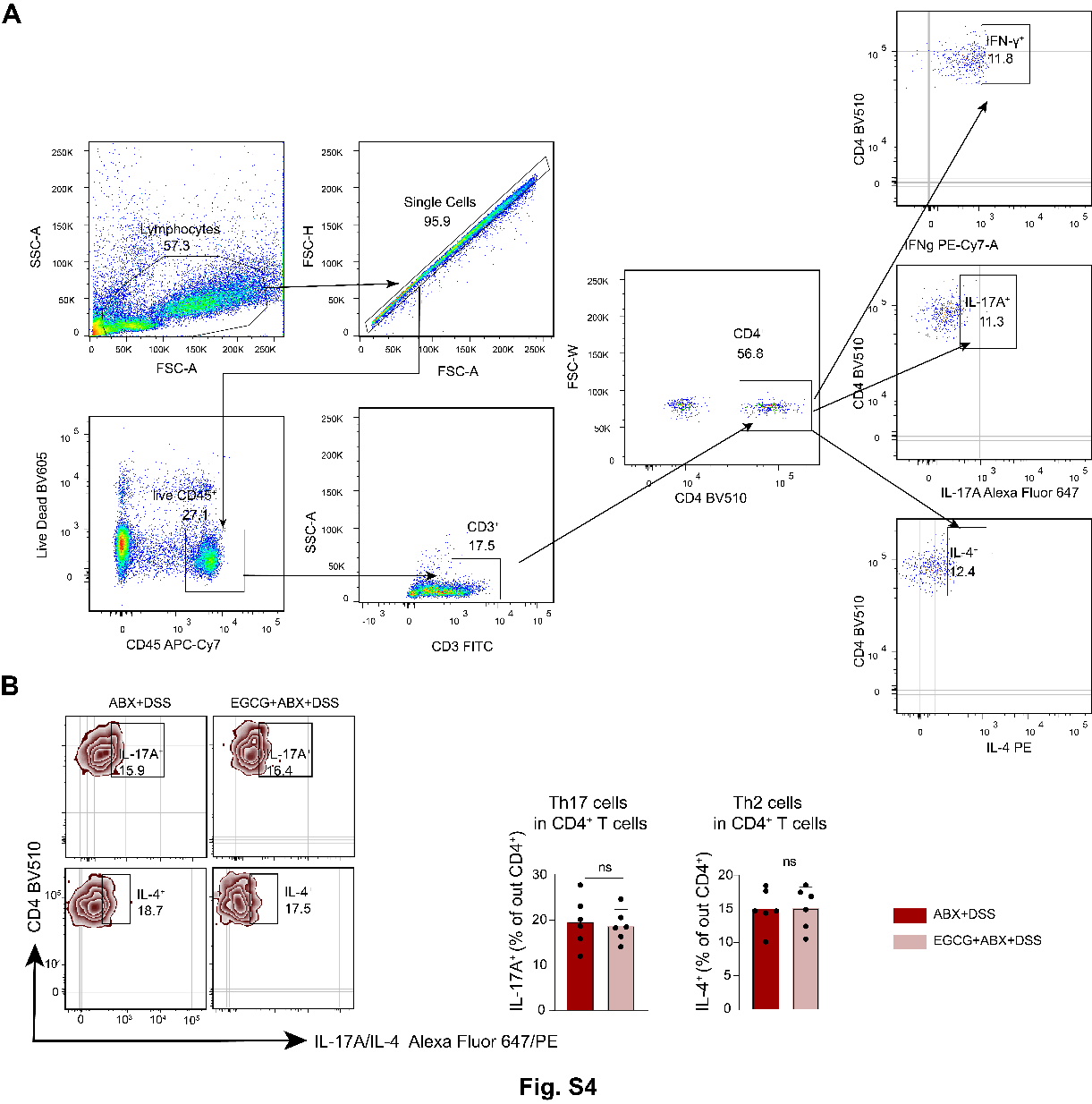


**Figure S4, related to Figure 2.** **EGCG inhibits DSS‑induced Th1 polarization in mice.**

Mice were administered EGCG at 80 mg/kg/day orally for 21 days, then treated with an antibiotic cocktail (ABX, a volume of 10μL/g body weight of drinking water supplemented with 0.1 mg/mL Amphotericin-B, 10 mg/mL Ampicillin, 10 mg/mL Neomycin Trisulfate salt hydrate, 10mg/mL Metronidazole, and 5 mg/mL Vancomycin hydrochloride) for 2 weeks. The mice were then given water containing 2.5% (w/v) DSS for 5 days, allowed for 2 days recovery with normal water.

(A) Gating strategy for isolated colonic T cell subsets.

(B) The colon cells were dissociated and Th subsets were detected and quantified. Data are one representative of two independent experiments. n = 6. Data are presented as mean ± SEM. Statistical significance was determined using unpaired Student’s t-test. * *P* ≤ 0.05, ** *P* ≤ 0.01.


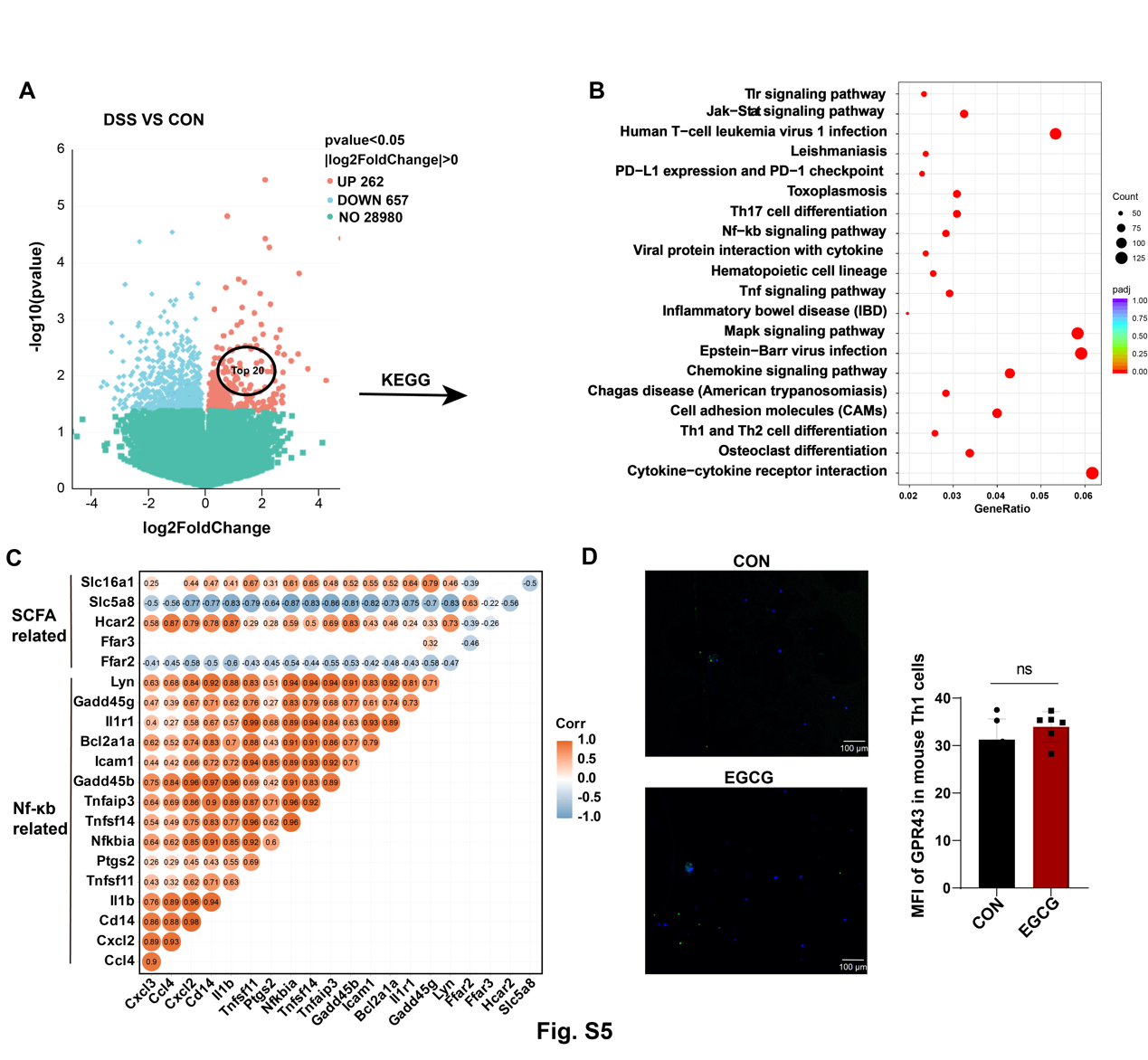


**Figure S5, related to Figure 3.** **EGCG activates colonic GPR43 and inhibits DSS-induced Nf-κb signaling pathway in mice.**

Mice were administrated 2.5% (w/v) DSS in drinking water for 5 days after they were treated with 80 mg/kg/day EGCG via oral gavage for 21 days, then allowed to recover for 2 days using normal drinking water.

(A and B) RNA-seq was performed to analyze gene expression in colon. Compared with the CON group, the up-regulated DEGs (A) in the DSS group were analyzed, and the top 20 signaling pathways were displayed by KEGG enrichment (B). n = 8-10.

C, Spearman’s analysis was applied to measure the correlation between Nf-κb signaling pathway involved genes and SCFAs related genes in colon in EGCG+DSS group. n = 8-10.

D, Naïve CD4^+^ T cells were isolated and sorted from mouse spleen, then treated with 1.0 μM EGCG for 24 h after incubated with anti-CD3, anti-CD28, and Th1 polarization proteins for 3 days, and expression of GPR43 protein were detected at the fourth day. Data are one representative of two independent experiments. n = 6. Data were analyzed with unpaired t-test and represented with mean ± SEM.

**
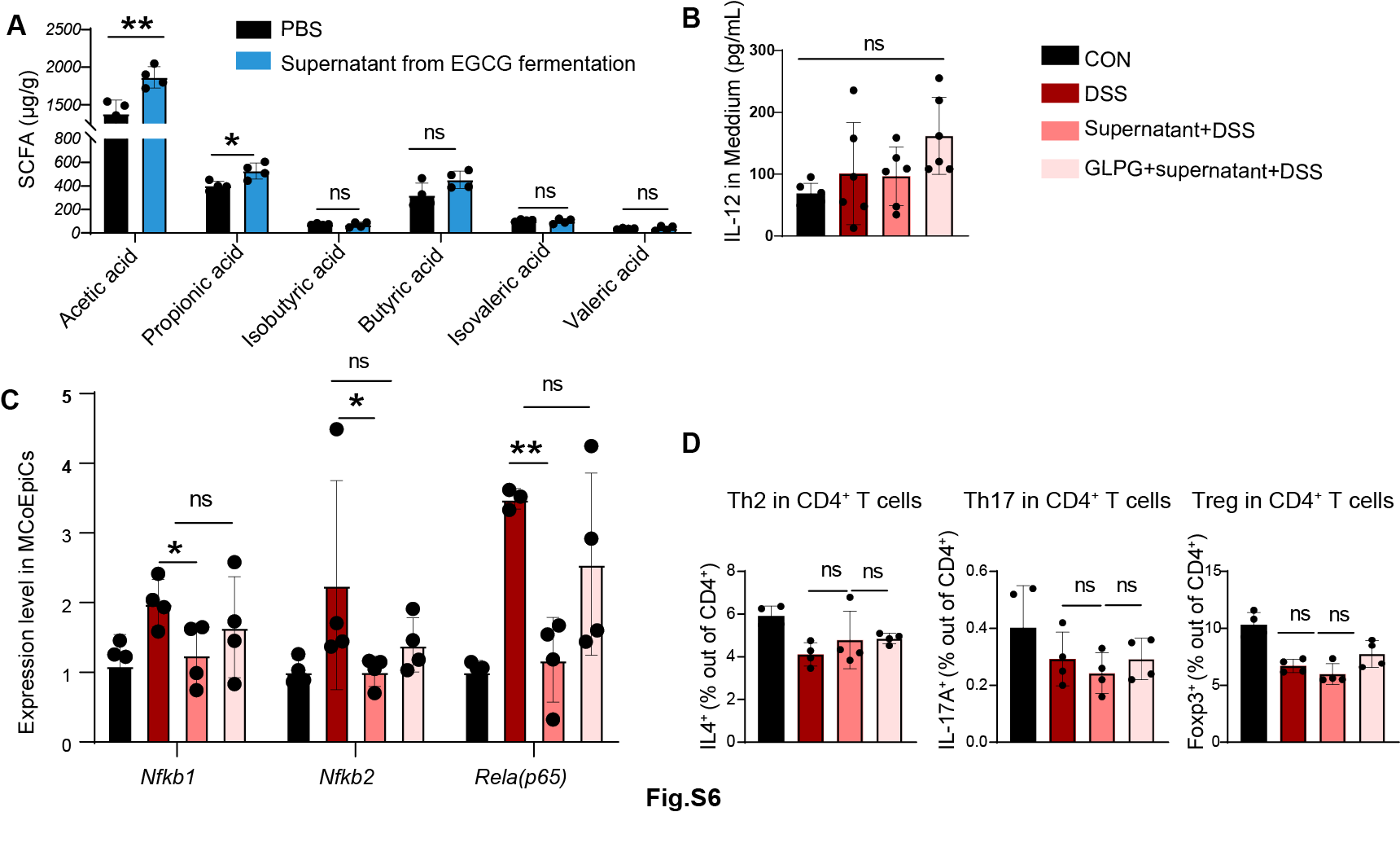
**

**Figure S6, related to Figure 5. Fermentation from EGCG and colonic contents promotes the production of SCFA and inhibits the NF-κB signaling pathway of mouse colonic epithelia.**

(A) Mice were given oral EGCG for 21days for *in vivo* fermentation, and colon contents were collected. EGCG (2 mg) was dissolved in 1 mL of PBS and was added to the colon contents for *in vitro* fermentation under anaerobic conditions for 48 hours. The same volume of PBS was added as a control. After fermentation, a gas chromatograph (GC) system was used to detect the SCFA concentrations. Data are one representative of two independent experiments. n = 4.

The fermentations were centrifuged, then concentrated at low temperature and diluted with cell culture medium. The resulting supernatant was used to treat mouse colonic epithelial cells (MCoEpiCs) for 24 hours after epithelial cells were treated with GLPG for GPR43 inhibition. Then culture medium was refreshed and followed by 2.5% (w/v) DSS treatment. The culture medium was refreshed to exclude previous treatment subjects, and cells were cultured for another 24 hours. Subsequently, the resulting medium was collected to treat the activated mouse T cells, which were isolated from spleen and stimulated with anti-CD28 and anti-CD3.

(B-D)

(B) IL-12 in the Medium. Data are one representative of two independent experiments. n = 6.

(C) NF-κB-related genes in MCoEpiCs were detected. Data are one representative of two independent experiments. n = 4.

(D) Effector Tell subtypes including Th2, Th17, and Treg were detected by flow cytometry. Data are one representative of two independent experiments. n = 4.


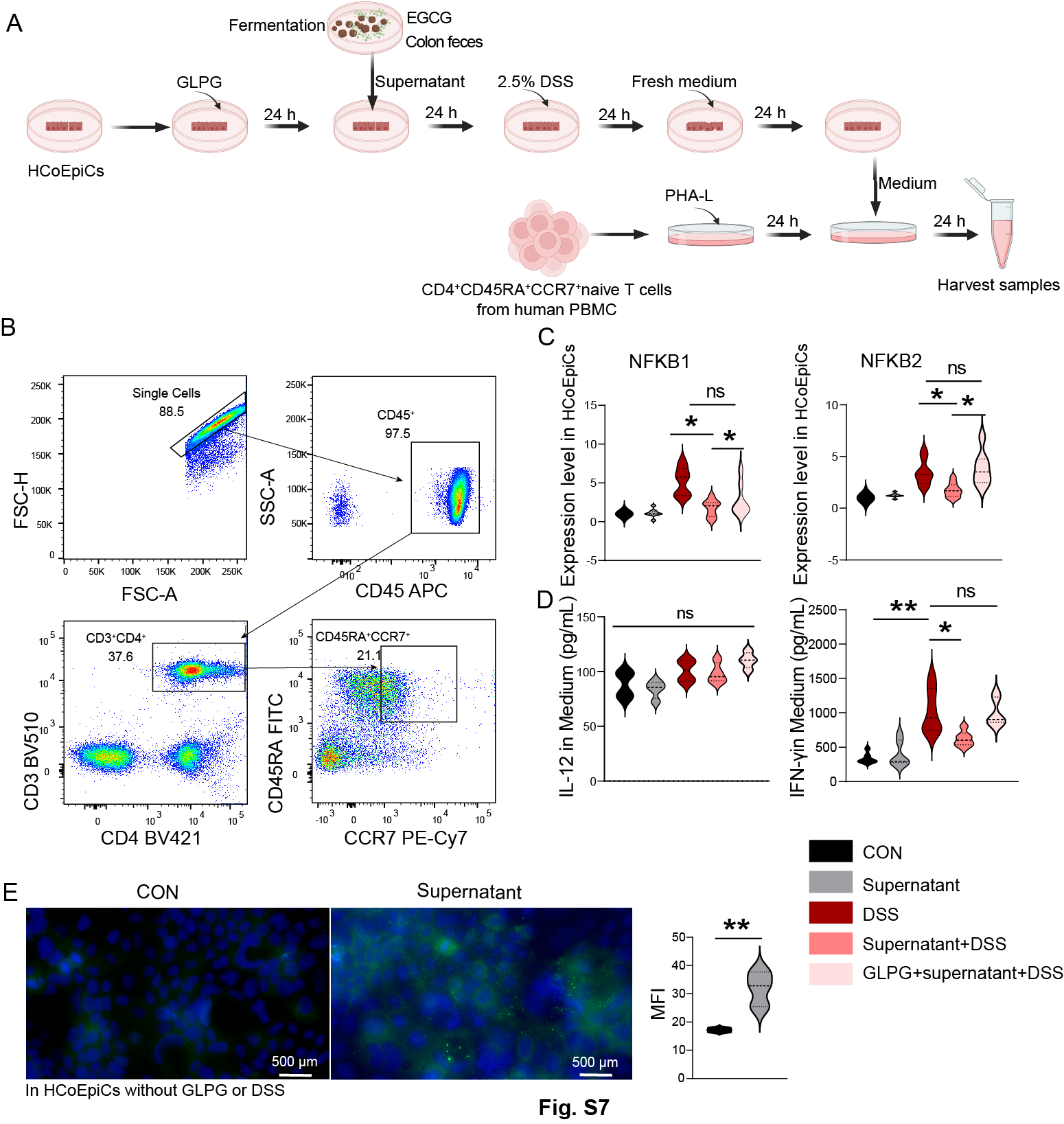


**Figure S7, related to Figure 6.** **EGCG fermentation increases GPR43 expression in HCoEpiCs.**

(A) Experimental scheme for (B-D). Mice were given oral EGCG for 21days for *in vivo* fermentation, and colon contents were collected. EGCG (2 mg) was dissolved in 1 mL of PBS and was added to the colon contents for *in vitro* fermentation under anaerobic conditions for 48 hours. The same volume of PBS was added as a control. After fermentation, a gas chromatograph (GC) system was used to detect the SCFA concentrations. The fermentations were centrifuged, then concentrated at low temperature and diluted with cell culture medium. The resulting supernatant was used to treat human colonic epithelial cells (HCoEpiCs) for 24 hours after HCoEpiCs were treated with GLPG. Then culture medium was refreshed and followed by 2.5% (w/v) DSS treatment. The culture medium was refreshed and epithelial cells were cultured for another 24 hours. Subsequently, the above Medium was collected to treat the activated human CD4+ T cells, which were isolated from PBMC and stimulated with PHA-L.

(B) Gating strategy for sorting naïve CD4^+^ T cells from human PBMC.

(C) NF-κB related genes in EGCG fermentation treated or nontreated HCoEpiCs were detected by qRT-PCR. Data are one representative of two independent experiments. n = 4.

(D) IL-12 and IFN-γ in cell culture medium were determined by ELISA. Data are one representative of two independent experiments. n = 4.

(E) GPR43 expression in HCoEpiCs were tested using immunofluorescence assay, representative images were shown, and mean fluorescence density was calculated. Data are one representative of two independent experiments. n = 4.

Data are presented as mean ± SEM. Statistical significance was determined using one-way ANOVA, followed by Tukey’s multiple-comparison tests or Student’s t-test. * *P* ≤ 0.05, ** *P* ≤ 0.01.

**TableS1 Key resources table**

| **REAGENT or RESOURCE** | **SOURCE** | **IDENTIFIER** |
| --- | --- | --- |
| **Antibodies for flow cytometry** | | |
| APC-Cy™7 Hamster anti-Mouse CD3ε Antibody | BD Pharmingen | # 557596 |
| PerCP-Cy™5.5 Rat anti-Mouse CD4 Antibody | BD Pharmingen | # 550954 |
| PerCP-Cy5.5 Rat Anti-Mouse CD4 Antibody | BD Pharmingen | # 569923 |
| FITC Rat anti-Mouse CD8a Antibody | BD Pharmingen | # 553030 |
| APC Rat Anti-Mouse CD8b Antibody | BD Pharmingen | # 569870 |
| BV421 Rat anti-Mouse F4/80 Antibody | BD Pharmingen | # 565411 |
| Alexa Fluor® 647 Mouse anti-Rat IFN-γ Antibody | BD Pharmingen | # 562213 |
| PE-Cy™7 Rat anti-Mouse IFN-γ Antibody | BD Pharmingen | # 557649 |
| Alexa Fluor® 647 Rat anti-Mouse IL-17A Antibody | BD Pharmingen | # 560184 |
| PE Rat anti-Mouse IL-4 Antibody | BD Pharmingen | # 554435 |
| PE Mouse anti-Mouse Foxp3 Antibody | BD Pharmingen | # 566881 |
| FITC Mouse anti-Human CD45RA Antibody | BD Pharmingen | # 555488 |
| PE-Cy7 Rat anti-Human CCR7 (CD197) Antibody | BD Pharmingen | # 557648 |
| BV510 Mouse anti-Human CD3 Antibody | BD Pharmingen | # 564713 |
| BV421 Mouse anti-Human CD4 Antibody | BD Pharmingen | # 562424 |
| APC-H7 Mouse anti-Human CD45 Antibody | BD Pharmingen | # 560178 |
| APC Mouse anti-Human IFN-γ Antibody | BD Pharmingen | # 554702 |
| BV786 Rat anti-Human IL-4 Antibody | BD Pharmingen | # 564113 |
| PE Mouse anti-Human IL-17A Antibody | BD Pharmingen | # 560486 |
| PE Mouse anti-Human FoxP3 Antibody | BD Pharmingen | # 560046 |
| APC Rat anti-Mouse CD45 Antibody | BD Pharmingen | # 559864 |
| FITC Hamster anti-Mouse CD3ε Antibody | BD Pharmingen | # 553061 |
| BV510 Mouse anti-Human CD4 Antibody | BD Pharmingen | # 562971 |
| PE-Cy™7 Rat anti-Mouse CD62L Antibody | BD Pharmingen | # 560516 |
| PE Rat anti-Mouse CD44 Antibody | BD Pharmingen | # 553134 |
| Alexa Fluor® 647 anti-mouse CD8b Antibody | BioLegend | # 126612 |
| APC/Cyanine7 anti-mouse CD3ε Antibody | BioLegend | # 100330 |
| PE anti-mouse CD79a (Igα) Antibody | BioLegend | # 133103 |
| APC Rat anti-Mouse CD8a Antibody | Proteintech | # APC-65069 |
| FITC Rat anti-Mouse CD4 Antibody | Proteintech | # FITC-65104 |
| PE Rat anti-Mouse IFN gamma Antibody | Proteintech | # PE-65153 |
| PE-Cy™7 anti-Mouse/Rat IL-17A Antibody | eBioscience | # 25-7177-82 |
| PE anti-mouse CD79a (Igα) Antibody | BioLegend | # 133103 |
| **FACS Reagent** | | |
| Cytofix/Cytoperm™ Fixation/Permeabilization Kit | BD Pharmingen | # 554714 |
| Transcription Factor Buffer Set | BD Pharmingen | # 562574 |
| Protein transport inhibitor | BD Pharmingen | # 555029 |
| Cell actvition/polarization Reagent |  |  |
| Phytohemagglutinin-L | Roche | # 11249738001 |
| Purified anti-mouse CD3ε Antibody | BioLegend | # 100340 |
| Purified anti-mouse CD28 Antibody | BioLegend | # 102116, |
| Purified anti-mouse IL-4 Antibody | BioLegend | # 504122 |
| Recombinant Mouse IL-12 | BioLegend | # 577002 |
| Recombinant Mouse IL-2 | BioLegend | # 575402 |
| **Primary antibodies for Western blot or Immunofluorescence** | | |
| Anti-p-p65 Antibody | Abcam | # ab31624 |
| Anti-p105/50 Antibody | Abcam | # ab32360 |
| Anti-IL-12A Antibody | Abcam | # ab133751 |
| Anti-IL-12B Antibody | Abcam | #ab133752 |
| Anti-Beta Actin antibody | Abcam | # ab8227 |
| Anti-Histone H3 antibody | Abcam | # ab18521 |
| Anti-GPR43 Antibody | Absin | # abs122668 |
| Anti-GPR43 Antibody | Santa Cruz | # sc-32906 |
| **Antibiotics** | | |
| Amphotericin B | Santa Cruz | # 1397-89-3 |
| Ampicillin | Sigma-Aldrich | # 7177-48-2 |
| Neomycin trisulfate salt hydrate | Sigma-Aldrich | # 1405-10-3 |
| Metronidazole | Sigma-Aldrich | # 443-48-1 |
| Vancomycin hydrochloride | Sigma-Aldrich | # 1404-93-9 |
| **Oligonucleotides** | **Gene names** |  |
| CTCCCTTGGATCTGAGCTGG | *Il12a-F* |  |
| GTTGGAACGCTGACCATAGAG | *Il12a-R* |  |
| TGGGAGTACCCTGACTCCTG | *Il12b-F* |  |
| AGGAACGCACCTTTCTGGTT | *Il12b-R* |  |
| AGACAATCAGGCCATCAGCAA | *Ifng-F* |  |
| TGTGGGTTGTTGACCTCAAACT | *Ifng-R* |  |
| GTGTCCGTGGAGTGGACG | *Il12rb1-F* |  |
| GCACGAGCCACTCTGACT | *Il12rb1-R* |  |
| GCAGCATCTTCTAAGCCCTG | *Lta-F* |  |
| ATGTGGAGAACCTGCTGCTG | *Lta-R* |  |
| AAGCCATGTACCTTGAGGTTAGT | *Cxcr3-F* |  |
| TCAGGCTGAAATCCTGTGGG | *Cxcr3-R* |  |
| AGCAACCACCTGTTGTGGTC | *Tbx21-F* |  |
| GGGAACATTCGCCGTCCTT | *Tbx21-R* |  |
| TCGTTCAAGCGTGTCCTGG | *Stat4-F* |  |
| GCAGATGCCGGATTTCCATAG | *Stat4-R* |  |
| GTCATCCCGCAGAGAGAACG | *Stat1-F* |  |
| GCAGAGCTGAAACGACCTAGA | *Stat1-R* |  |
| GCTGTCATCGATTTCTCCCCT | *Il10-F* |  |
| GACACCTTGGTCTTGGAGCTTAT | *Il10-R* |  |
| CTCTGGCACAGAAGTTGGGT | *Nfkb1-F* |  |
| ATCCCGGAGTTCATCTCATAGT | *Nfkb1-R* |  |
| GGTCTCCATGAGGGAGTCAGG | *Nfkb2-F* |  |
| CACTGTCTTCTTTCACCTCTGTGC | *Nfkb2-R* |  |
| ACCCTGACCATGGACGATCT | *Rela(p65)-F* |  |
| GTTGCTTCGGCTGTTCGATG | *Rela(p65)-R* |  |
| CTTGCCCAAGGAGAATGACC | *Ffar2-F* |  |
| GCAGGGAGCCCAGTAAGAAAG | *Ffar2-R* |  |
| TGCGGCATGTTCTGGATTTG | *Il2-F* |  |
| CAAATGTGTTGTCAGAGCCCTTT | *Il2-R* |  |
| TGGAAAGGAGCTGATTGCTGT | *Fabp2-F* |  |
| TCCTTCATATGTGTAGGTCTGGA | *Fabp2-R* |  |
| CGCTCTCGCTCATCTTCGAT | *Dhcr24-F* |  |
| TCCATTCCCGGACCTGTTTC | *Dhcr24-R* |  |
| CTGGCAACACAGATGGAGGA | *Il2r-F* |  |
| TGGCGTCTCAGATTTGGCTT | *Il2r-R* |  |
| AGAAGAGCACCCCAGAAAGC | *Cd44-F* |  |
| TTGTGGGCCGAAGTGGTG | *Cd44-R* |  |
| GCTGGGACATCACAGAAGGT | *Il1r2-F* |  |
| CACCCACATCTACTGAATCTGGA | *Il1r2-R* |  |
| CCTTCAGAGACTGTGCGGAA | *Icos-F* |  |
| TCCATGTCACAGTAGGCCTTG | *Icos-R* |  |
| AGCCTGCTCTAGGGGTGTCA | *Angpt2-F* |  |
| CAATTCCACCAGCACGTCAC | *Angpt2-R* |  |
| GGGGGCCCAGGCCATTA | *Rac2-F* |  |
| TGAGGCCGAACTTCTCACAG | *Rac2-R* |  |
| TGCAGCAGACCAAGGAGATG | *Vav1-F* |  |
| TGCATTGGGGGCTTTACTGT | *Vav1-R* |  |
| TAGAGGAGGGAGTGAGGCTG | *NFKB1-F* |  |
| TTCCTTCACCTCTGCTGTGC | *NFKB1-R* |  |
| **Software and algorithms** |  |  |
| Graphpad Prism 8 | GraphPad  Software | [www.graphpad.com](http://www.graphpad.com) |
| FlowJo v10 | FlowJo | [www.bdbiosciences.com](http://www.bdbiosciences.com) |
| ZEN 3.3 | Zeiss | [www.zeiss.com](http://www.zeiss.com) |
| ImageJ | NIH | [www.imagej.nih.gv](http://www.imagej.nih.gv) |
